# Anisotropic Plasmonic Metal Heterostructures as Theranostic Nanosystems for Near Infrared Light‐Activated Fluorescence Amplification and Phototherapy

**DOI:** 10.1002/advs.201900158

**Published:** 2019-04-05

**Authors:** Yun Chang, Yanlin Feng, Yan Cheng, Runxiao Zheng, Xiaqing Wu, Hui Jian, Dawei Zhang, Zhaohui Tang, Zhenxin Wang, Jiaming Hao, Haiyuan Zhang

**Affiliations:** ^1^ Laboratory of Chemical Biology Changchun Institute of Applied Chemistry Chinese Academy of Sciences Changchun 130022 China; ^2^ University of Chinese of Academy of Sciences Beijing 100049 China; ^3^ University of Science and Technology of China Anhui 230026 China; ^4^ Key Laboratory of Polymer Ecomaterials Changchun Institute of Applied Chemistry Chinese Academy of Sciences Changchun 130022 China; ^5^ State Key Laboratory of Electroanalytical Chemistry Changchun Institute of Applied Chemistry Chinese Academy of Sciences Changchun 130022 China; ^6^ State Key Laboratory of Infrared Physics Shanghai Institute of Technical Physics Chinese Academy of Sciences Shanghai 200083 China

**Keywords:** anisotropic heterostructures, fluorescence amplification, hot electrons, phototherapy, theranostics

## Abstract

The development of sophisticated theranostic systems for simultaneous near infrared (NIR) fluorescence imaging and phototherapy is of particular interest. Herein, anisotropic plasmonic metal heterostructures, Pt end‐deposited Au nanorods (PEA NRs), are developed to efficiently produce hot electrons under 808 nm laser irradiation, exhibiting the strong electric density. These hot electrons can release the heat through electron‐phonon relaxation and form reactive oxygen species through chemical transformation, as a result of potent photothermal and photodynamic performance. Simultaneously, the confined electromagnetic field of PEA NRs can transfer energy to adjacent polyethylene glycol (PEG)‐linked NIR fluorophores (CF) based on their energy overlap mechanism, leading to remarkable NIR fluorescence amplification in CF‐PEA NRs. Various PEG linkers (1, 3.4, 5.0, and 10 kD) are employed to regulate the distance between CF and PEA NRs of CF‐PEA NRs, and the maximum fluorescence intensity is achieved in CF_5k_‐PEA NRs. After further attachment with i‐motif DNA/Nrf2 siRNA chimera to simultaneously suppress both cellular antioxidant defense and hyperthermia resistance effects, the final biocompatible CF_5k_‐*b*PEA@siRNA NRs present promising NIR fluorescence imaging ability and 808 nm laser‐activated photothermal and photodynamic therapeutic effect in MCF7 cells and tumor‐bearing mice, holding great potential for cancer therapy.

Near infrared (NIR) light‐activated theranostic strategy has received considerable attention due to its simultaneous performance of cancer diagnosis and therapy in the deep tissue.^1^ Sophisticated nanosystems have been usually designed as theranostic platforms to pursue noninvasive detection and treatment, spatiotemporal and remote controllability, and excellent selectivity and biocompatibility, where the combination of NIR fluorescence imaging and NIR light‐triggered phototherapeutic treatment (photothermal therapy (PTT) and photodynamic therapy (PDT)) is of great interest.^2^ However, the combined PTT and PDT process is usually required to integrate distinct photosensitizer agent (PSA) and photothermal agent (PTA) in a single nanosystem,^3^ causing great inconvenience; NIR fluorophores usually show a low quantum yield, leading to the diminished fluorescence intensity.^4^ All these disadvantages of PSA and PTA as well as NIR fluorophores dramatically limit the application of NIR fluorescence image‐guided photothermal and photodynamic combination therapy.

Anisotropic plasmonic metal heterostructures are emerging as potent theranostic platforms to improve the fluorescence imaging and phototherapeutic effect of cancer based on their unique electronic properties. It has been known that localized surface plasmon resonance (LSPR) excited by the incident light can extremely promote the electromagnetic field enhancement and energetic charge carrier generation in plasmonic nanostructures.^5^ On one hand, the enhanced electromagnetic field can transfer energy to the fluorophore that is located in close proximity to plasmonic nanostructures, leading to significantly amplified fluorescence intensity of fluorophore when the LSPR band of plasmonic nanostructures overlaps with the fluorophore's absorption or emission band and the distance between plasmonic nanostructures and fluorophores is appropriate (Scheme S1, Supporting Information).^6^ On the other hand, the generated energetic charges can promote the reactive oxygen species (ROS) generation through both chemical and energy transformation process,^7^ and release the heat through electron‐phonon relaxation process,^8^ endowing the plasmonic nanostructures with the photodynamic and photothermal performance (Scheme S2, Supporting Information). Furthermore, the electromagnetic field‐mediated fluorescence enhancement and energetic carriers‐induced photodynamic and photothermal performance can exhibit the similar ascending tendency. More importantly, the well‐designed anisotropic architecture of nanosystems can further facilitate the charge carrier spatial separation based on the Fermi level alignment on disparate metals,^9^ leading to more remarkable hot electron generation and subsequently more effective photothermal and photodynamic performance.

In the present study, Pt dots were anisotropically grown at both ends of gold nanorods (Au NRs) to form Pt end‐deposited Au nanorods (PEA NRs) with the LSPR maximum at 808 nm, which possess excellent plasmonic property, metal heterostructure, and anisotropic architecture. Upon 808 nm laser irradiation, an efficient electron–hole spatial separation along longitudinal axis of rods can occur in PEA NRs because of their end‐deposition morphology, leading to more significant hot electron generation in PEA NRs compared with Pt body‐deposited gold nanorods (PBA NRs) or traditional Au@Pt core–shell nanorods where the electron–hole separation is not efficient due to the homogeneous coating and fast recombination. The generated hot electron can release heat based on electron‐phonon relaxation and form ROS (singlet oxygen (^1^O_2_), superoxide radical (O_2_^•−^), and hydroxyl radical (•OH)) based energy and chemical transformation process, affording photothermal and photodynamic performance (**Figure** **1**a). CF790, a NIR fluorophore, was engineered on the surface of PEA NRs through a polyethylene glycol (PEG) linker to form CF‐PEA NRs, which enables the emission maximum (806 nm) of CF790 matching with the LSPR absorption maximum (808 nm) of PEA NRs, making it possible for the electromagnetic field‐facilitated NIR fluorescence amplification.^10^ The distance between PEA NRs and CF790 was further optimized through screening molecular weights of PEG linkers in a broad range, achieving the maximum NIR fluorescence intensity (Figure 1b). Given that cells in response to photodynamic therapy and photothermal therapy can usually trigger phase II enzyme and heat shock protein (HSP70) expression through Nrf2 activation (Scheme S3, Supporting Information) to reduce the oxidant attacking and hyperthermia effect,^11^ an i‐motif DNA/Nrf2 siRNA duplex chimera was conjugated to the surface of CF‐PEA NRs to form CF‐PEA@siRNA NRs for silencing Nrf2 gene, aiming at simultaneously reducing phase II enzyme and HSP70 expression.^12^ In response to acidic stimuli (pH 4.5–5.5), the i‐motif segment of this DNA/RNA chimera can be allosterically converted from single strand to quadruplex (Figure 1c),^13^ capable of releasing Nrf2 siRNA to attenuate antioxidant defense and hyperthermia resistance, as a result of creating favorable cellular environment for strengthening both PDT and PTT effects. To improve the biocompatibility, prolong the in vivo circulation time, enhance the tumor cell penetration ability, and facilitate the endosomal disruption, CF‐PEA@siRNA NRs were fabricated with a pH‐responsive and charge reversible cell‐penetrating peptide (CPP)^14^ through PEG linkers (Figure 1c).

**Figure 1:**
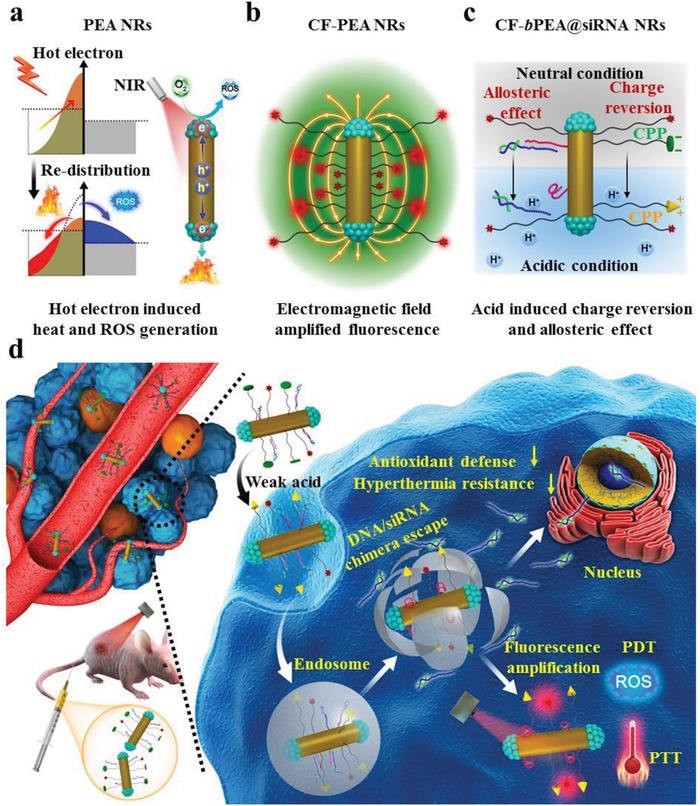
Illustrating the theranostic application of CF‐*b*PEA@siRNA NRs. a) NIR laser‐activated charge carrier spatial separation along the longitudinal axis of PEA NRs, as a result of dense hot electron generation that can promote ROS production through energy and chemical transformation process and release the heat through electron‐phonon relaxation process. b) Enhanced electromagnetic field of PEA NRs can transfer energy to the adjacent CF790 in appropriate distance when both energies match, leading to significantly amplified NIR fluorescence of CF‐PEA NRs. c) i‐motif DNA/Nrf2 siRNA chimera on CF_5k_‐*b*PEA@siRNA NRs can allosterically release Nrf2 siRNA in response to the acidic condition (pH 4.5–5.5) to suppress cellular antioxidant defense and hyperthermia resistance effect; acid (pH 6.5)‐responsive and charge reversible cell‐penetrating peptides (CPP) on CF_5k_‐*b*PEA@siRNA NRs can improve the biocompatibility, prolong the in vivo circulation time, enhance the tumor cell penetration ability, and facilitate the endosomal disruption. d) CF_5k_‐*b*PEA@siRNA NRs can exhibit promising NIR fluorescence imaging performance as well as PDT and PTT effect after intravenous administration to tumor‐bearing mice.

This sophisticated nanosystem, CF‐*b*PEA@siRNA NRs, was designed as a theranostic platform for NIR fluorescence imaging and phototherapy of cancer. As shown in Figure 1d, once intravenously administered to tumor‐bearing mice, negatively charged CF‐*b*PEA@siRNA NRs were expected to realize a long circulation time and high accumulation at the tumor region based on enhanced permeability and retention (EPR) effect. Biodistribution and tumor accumulation of CF‐*b*PEA@siRNA NRs could be visualized through significantly amplified CF790 NIR fluorescence. The weak acidic microenvironment (pH 6.5) of tumor could initiate the charge reversal of CPP from negative to positive, beneficial for the cell uptake of CF‐*b*PEA@siRNA NRs and following endosomal disruption. The released Nrf2 siRNA from CF‐*b*PEA@siRNA NRs in acidic endosomal compartment (pH 4.5–5.5) could attenuate the cellular hyperthermia resistance and antioxidant performance, aiming to optimize the cellular microenvironment for PTT and PDT. Upon 808 nm laser irradiation, the combined PTT and PDT effects of CF‐*b*PEA@siRNA NRs could significantly inhibit the tumor growth.

Au NRs were prepared through a seed‐mediated method.^15^ With addition of 5‐bromosalicylic acid that selectively blocks the side of Au NRs to inhibit Pt deposition, the platinum precursor (H_2_PtCl_6_) preferred to be deposited at the ends of Au NRs to form PEA NRs.^15^, ^16^ Once Pt was nucleated on the rods, there was a thermodynamic preference for additional Pt growth on Pt rather on the exposed Au surface because of the higher cohesive and surface energies of Pt.^17^ To better elucidate the advantage of PEA NRs, PBA NRs with homogeneous Pt deposition on Au NRs were prepared in a similar process without addition of 5‐bromosalicylic acid. **Figure** **2**a and Figure S1a (Supporting Information) shows the transmission electron microscopy (TEM) images of PEA, PBA, and Au NRs. Au NRs had homogeneous sizes of 42.3 ± 4.2 nm in length and 12.6 ± 2.1 nm in diameter. Pt exhibited the island growth pattern (Volmer–Weber model) on PEA and PBA NRs,^18^ where PEA NRs showed Pt deposition at both ends of Au NRs while PBA NRs displayed the homogeneous Pt deposition over the whole body of Au NRs. The elemental mapping (Figure 2b) confirmed that PEA NRs had more evident Pt deposition at the ends of Au NRs than that at the lateral sides, while PBA NRs showed a more even Pt distribution at the ends and sides of Au NRs. X‐ray diffraction (XRD) patterns (Figure S1b, Supporting Information) further revealed the typical {111}, {100}, and {110}) peaks of Pt existing in both PEA and PBA NRs, confirming the Pt deposition on Au NRs. Ultraviolet (UV)–visible (vis) near infrared spectra indicated Pt deposition on Au NRs could redshift the LSPR peak of Au NRs from 740 to 808 nm (Figure 2c and Figure S1c, Supporting Information).^19^

**Figure 2:**
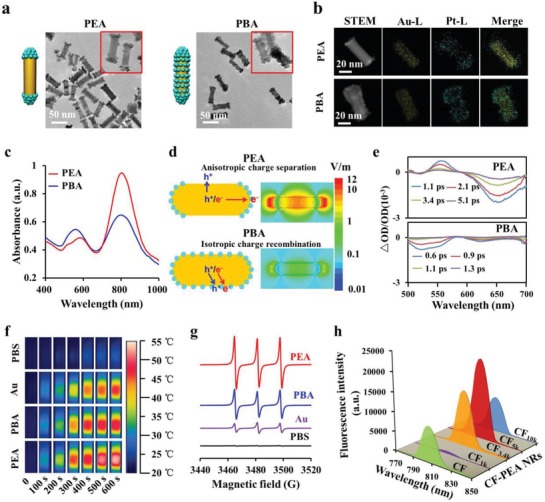
Physicochemical characterization of PEA and PBA NRs. a) TEM images. b) Scanning transmission electron microscopy (STEM) images and energy dispersive X‐ray spectroscopy (EDS) elemental mapping images. c) UV–vis NIR absorption spectra. d) Spatial distribution of enhanced electric field at LSPR excitation based on FDTD simulation. e) Transient absorption spectra of PEA and PBA NRs at representative delay time. f) Infrared thermal images of NR aqueous suspension (equivalent to 50 µg mL^−1^ Au) irradiated by an 808 nm laser (0.75 W cm^−1^) for 600 s. g) ESR spectra of TEMP‐^1^O_2_ spin adducts were generated by Au, PBA, and PEA NRs (equivalent to 25 µg mL^−1^ Au) aqueous solution containing 0.3 mol L^−1^ TEMP under 808 nm laser irradiation (0.75 W cm^−2^, 10 min); h) NIR fluorescence emission spectra of free CF and various CF‐PEA NRs with 1, 3.4, 5, and 10 kD of PEG linkers at excitation of 784 nm.

Furthermore, finite difference time domain (FDTD) simulation (Figure 2d) theoretically presented the spatial distribution of electric intensity in PEA and PBA NRs, where the more significant electric field enhancement occurred at the ends of PEA NRs compared with those of PBA NRs. Transient absorption (TA) spectroscopy measurements experimentally detected the generation and dynamics of energetic charge carries in PEA and PBA NRs,^20^ corroborating the higher hot electron generation efficiency (Figure 2e) and slower charge decay speed (Figure S2, Supporting Information) of PEA NRs than PBA NRs, which is ascribed to the more effective electron–hole spatial separation efficiency of PEA NRs. Taken together, PEA NRs can produce strong electric field density and abundant hot electrons. The abundant hot electrons are beneficial for promoting the reactive oxygen species generation through both chemical and energy transformation process, and also for releasing the heat through electron‐phonon relaxation process (Scheme S2, Supporting Information).

The significantly enhanced hot electron density of PEA NRs can promote photothermal and photodynamic performance. Photothermal performance of PEA, PBA, and Au NRs was investigated by comparing their temperature elevation profiles under 808 nm laser irradiation (0.75 W cm^−2^, 10 min). Figure S3 (Supporting Information) shows PEA NRs could induce the most potent photothermal activity with a photothermal conversion efficiency of 55.4% (Figure S4a,b, Supporting Information), followed by PBA (52.9%) and Au NRs (49.3%), which could be clearly visualized by infrared thermal imaging (Figure 2f). The superior photothermal activity of PEA NRs compared with PBA NRs may be due to their higher hot electrons generation efficiency, which is beneficial for heat release through electron‐phonon relaxation.

The photodynamic performance of PEA, PBA, and Au NRs was also investigated under 808 nm laser irradiation (0.75 W cm^−2^, 10 min) through electron spin resonance (ESR) spectroscopy, and 2,2,6,6‐tetramethylpiperidine (TEMP, for ^1^O_2_) as well as 5,5‐dimethyl‐1‐pyrroline *n*‐oxide (DMPO, for O_2_^•−^ and •OH) were exploited as ROS‐sensitive trapping agent. Figure 2g and Figure S5a,b (Supporting Information) shows PEA NRs could induce the strongest ESR signals of ^1^O_2_, O_2_^•−^, and •OH, followed by PBA and Au NRs. Obviously, heterostructures (PEA and PBA NRs) can induce more efficient electron–hole separation than homostructures (Au NRs), and PEA NRs are more effective than PBA NRs, as a result of more ROS production. Moreover, both PEA and PBA NRs showed excellent photostability as demonstrated by Figure S6 (Supporting Information).

CF790, was attached to PEA NRs through PEG linker for enhancement of NIR fluorescence intensity. The LSPR absorption maximum (808 nm) of PEA NRs was very close to the fluorescence emission maximum (806 nm) of CF790, making it possible for the fluorescence amplification. To achieve the strongest NIR fluorescence, the distance between PEA NRs and CF790 was optimized by tuning the molecular weights of PEG linker (1, 3.4, 5, and 10 kDa), the length of which was estimated to be 4, 8, 10–20, and 25–30 nm, respectively.^21^ The very short distance can result in fluorescence quenching, whereas the very long distance will have no enhancement in the fluorescence intensity.[qv: 6a] The content of CF790 on various CF790‐PEG conjugated PEA NRs (CF‐PEA NRs) was controlled at the similar level (Figure S7a, Supporting Information),^22^ and the hydrodynamic sizes of CF‐PEA NRs became bigger and bigger with the increase of PEG molecular weight from 1k to 10k (Figure S7b, Supporting Information). Figure 2h shows that CF_1k_‐PEA NRs (CF790 was conjugated to PEA NRs through PEG linkers with the molecular weight of 1k) produced the reduced fluorescence intensity than free CF790 while CF_3.4k_‐PEA, CF_5k_‐PEA, and CF_10k_‐PEA NRs exhibited the enhanced intensity, where the enhancement of CF_5k_‐PEA NRs was the most remarkable. It means the appropriate PEG linker actually results in the largest NIR fluorescence amplification. Furthermore, the NIR fluorescence signal of CF_5k_‐PEA NRs could not be influenced by ROS (Figure S7c, Supporting Information), demonstrating the PDT process cannot affect the NIR fluorescence intensity.

The surface of CF_5k_‐PEA NRs was fabricated with thiol‐modified PEG_3.4k_‐CPP to form CF_5k_‐*b*PEA NRs with a hydrodynamic size of 222.4 ± 18.1 nm in culture medium for improving their biocompatibility, cellular uptake efficiency, and subsequent endosomal disruption. In vitro NIR fluorescence enhancement of CF_5k_‐*b*PEA NRs was examined in MCF‐7 cells. For comparison, other CF_1–10k_‐PEA NRs were similarly fabricated to form CF_1–10k_‐*b*PEA NRs. After 6 h of incubation with various CF_1–10k_‐*b*PEA NRs under weak acidic condition (pH 6.5), the cells showed similar dark‐field signal intensities (**Figure** **3**a), and Au element‐based inductively coupled plasma‐optical emission spectrometer (ICP‐OES) analysis (Figure S8a, Supporting Information) further confirmed the similar Au contents in these cells. In comparison, the cellular uptake at neutral conditions (Figure S8a, Supporting Information) was lower than those at acidic conditions, demonstrating the potential cell penetration ability of pH‐sensitive CPP under tumor acidic condition. However, NIR fluorescence microscopy (Figure 3b) revealed that the cells treated with CF_1k_‐*b*PEA NRs showed weaker NIR fluorescence compared with those treated with free CF790, while the cells treated with CF_3.4k_‐*b*PEA, CF_5k_‐*b*PEA, and CF_10k_‐*b*PEA NRs displayed the significantly enhanced NIR fluorescence, where CF_5k_‐*b*PEA NRs could result in the most prominent cellular fluorescence enhancement, which was further confirmed by flow cytometry analysis (Figure S8b, Supporting Information). This trend is in accordance with the profile of fluorescence emission spectra of various CF‐PEA NRs (Figure 2h), corroborating CF_5k_‐*b*PEA NRs can exhibit the highest cellular NIR fluorescence intensity.

**Figure 3:**
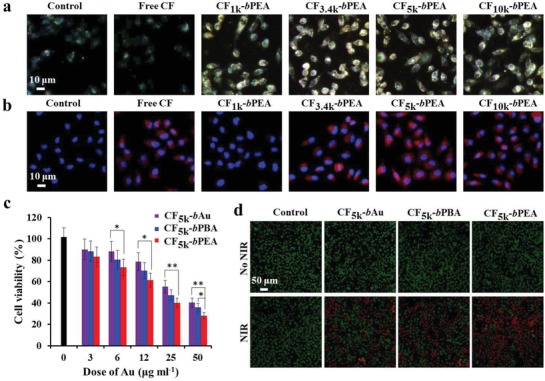
In vitro NIR fluorescence amplification and NIR laser activated phototherapeutic effect of CF_5k_‐*b*PEA NRs in MCF7 cells. a) Dark field images and b) NIR fluorescence images of MCF7 cells treated with free CF or various CF_1–10k_‐*b*PEA NRs (equivalent to 25 µg mL^−1^ Au) for 6 h. c) MTS viability assessment of MCF‐7 cells treated with different concentrations of CF_5k_‐*b*Au, CF_5k_‐*b*PBA, and CF_5k_‐*b*PEA NRs (according to Au content) for 24 h under 5 min of 808 nm laser irradiation (0.5 W cm^−2^). d) Live/dead cell staining of MCF‐7 cells treated with CF_5k_‐*b*Au, CF_5k_‐*b*PBA, and CF_5k_‐*b*PEA NRs (equivalent to 25 µg mL^−1^ Au) for 24 h with or without 5 min of 808 nm laser irradiation (0.5 W cm^−2^). *P < 0.05, **P < 0.01.

The phototherapeutic effect of CF_5k_‐*b*PEA NRs was assessed by 3‐(4,5‐Dimethylthiazol‐2‐yl)‐5‐(3‐carboxymethoxyphenyl)‐2‐(4‐sulfophenyl)‐2H‐tetrazolium (MTS) assay under 808 nm laser irradiation. For comparison, PEA and Au NRs were also similarly fabricated with CPP and CF790 to form CF_5k_‐*b*PBA and CF_5k_‐*b*Au NRs. After 6 h of treatment with various concentrations of these NRs in acidic culture medium (pH 6.5) followed by another 18 h of incubation in normal culture medium, the viability of MCF‐7 cells was found to be less affected (Figure S9a, Supporting Information), demonstrating the excellent biocompatibility of these NRs. However, when cells were treated with these NRs for 6 h in acidic culture medium followed by 5 min of 808 nm laser irradiation (0.5 W cm^−2^) and another 18 h of incubation in normal culture medium, both CF_5k_‐*b*PEA and CF_5k_‐*b*PBA NRs could more remarkably reduce the cell viability than CF_5k_‐*b*Au NRs (Figure 3c), where CF_5k_‐*b*PEA NRs could cause more severe reduction than CF_5k_‐*b*PBA NRs based on their more significant photothemral and photodynamic performance. Furthermore, calcein acetoxymethyl (AM)/propidium iodide Live/dead cell staining assay (Figure 3d) was carried out to further corroborate the biocompatibility and phototherapeutic effect of CF_5k_‐*b*PEA NRs in MCF‐7 cells. In comparison, all these NRs displayed lower phototherapeutic effects under neural condition (Figure S9b, Supporting Information), supporting pH‐sensitive CPP can improve the phototherapeutic efficiency of these NRs under acidic condition, which is potentially ascribed to their enhanced cellular uptake.

The effective PDT effect of CF_5k_‐*b*PEA NRs was ascribed to their large amount ROS production. However, since abiotic ROS can stimulate nuclear factor NF‐E2 related factor 2 (Nrf2) cellular signaling pathway to produce phase II enzymes responsible for antioxidant defense, the current NIR light‐activated oxidative injury induced by CF_5k_‐*b*PEA NRs probably has been dramatically attenuated.[qv: 11a,23] Moreover, the PTT effect of CF_5k_‐*b*PEA NRs can also enhance HSP70 expression, leading to cellular hyperthermia resistance. Thus, attenuating the cellular antioxidant defense and hyperthermia resistance potentially can potentiate the PDT and PTT effect. A thiol‐modified i‐motif DNA/Nrf2 siRNA chimera was immobilized on the surface of CF_5k_‐*b*PEA NRs to form CF_5k_‐*b*PEA@siRNA NRs (Figure S10, Supporting Information) for strengthening both PDT and PTT effect. As analyzed by agarose gel electrophoresis (**Figure** **4**a), CF_5k_‐*b*PEA@siRNA NRs could exhibit a pH‐responsive siRNA release behavior, suggesting that i‐motif DNA segment of CF_5k_‐*b*PEA@siRNA NRs can undergo the allosteric effect at a low pH environment, facilitating the Nrf2 siRNA chimera release. CF_5k_‐*b*PEA@siRNA NRs could also be effectively internalized into MCF‐7 cells (Figure S10c, Supporting Information). Confocal fluorescence microscopy further revealed that FITC‐labeled siRNA could colocalize with Lyso‐Tracker Red stained endosomes when cells were incubated with CF_5k_‐*b*PEA@FITC‐siRNA NRs (without CPP terminal) for 6 h, but could diffuse into the cytosol when cells incubated with CF_5k_‐*b*PEA@FITC‐siRNA NRs (with CPP terminal) (Figure 4b). This result confirms CF_5k_‐*b*PEA@siRNA NRs can efficiently release siRNA into cytosol due to CPP modification. Western blot analysis (Figure 4c) indicated that the Nrf2, HO‐1 (a typical phase II enzyme), and HSP 70 expression in cells treated with CF_5k_‐*b*PEA@siRNA NRs was much lower than those of cells treated with CF_5k_‐*b*PEA NRs, and furthermore, these Nrf2 expressions were inversely proportional to the increased concentration (12.5, 25, and 50 µg mL^−1^) of CF_5k_‐*b*PEA@siRNA NRs (Figure S11a, Supporting Information), and the Nrf 2 gene knockdown efficiency were 11.48%, 41.18%, and 66.27%, respectively (Figure S11b, Supporting Information), meaning Nrf2 siRNA has successfully taken effect.

**Figure 4:**
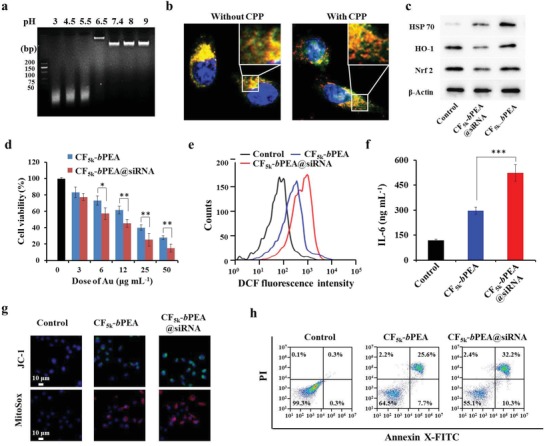
Strengthened phototherapeutic effect of CF_5k_‐*b*PEA@siRNA NRs compared with CF_5k_‐*b*PEA NRs in MCF‐7 cells. a) Gel electrophoresis analysis of siRNA releasing behavior from CF_5k_‐*b*PEA@siRNA NRs (equivalent to 50 µg mL^−1^ Au) under different pH buffers (pH 3.0, 4.5, 5.5, 6.5, 7.4, 8.0, and 9.0). b) Confocal microscopy images of cells treated with CF_5k_‐*b*PEA@FITC‐siRNA (equivalent to 25 µg mL^−1^ Au) with or without CPP attachment. Cells were stained with 2 µmol L^−1^ LysoTracker (red) and 1 µmol L^−1^ Hoechst 33258 (blue). c) Western blot analysis of Nrf2, HO‐1, and HSP 70 protein expression. d) MTS viability assessment of cells treated with different concentrations of CF_5k_‐*b*PEA@siRNA or CF_5k_‐*b*PEA NRs (according to Au content) for 24 h under 5 min of 808 nm laser irradiation (0.5 W cm^−2^). *P < 0.05, *P < 0.01, and *P < 0.001. e) Flow cytometry analysis of intracellular ROS level based on dichlorofluorescein (DCF) assay. f) ELISA to assess cellular IL‐6 secretion. g) Fluorescence microscopy images of cells stained by JC‐1 (green) or Mitosox Red (red) to detect membrane depolarization or mitochondrial superoxide generation. h) Flow cytometry analysis of apoptotic cells based on Annexin V‐FITC/PI assay. For (c) and (e–h), cells were treated with CF_5k_‐*b*PEA@siRNA or CF_5k_‐*b*PEA NRs (equivalent to 25 µg mL^−1^ Au) for 6 h, followed by 5 min of 808 nm laser irradiation (0.5 W cm^−2^) and another 18 h of incubation.

The downregulation of Nrf2 potentially can attenuate antioxidant defense and hyperthermia resistance capability, making cells more susceptible to ROS and heat injury as well as leading to enhanced NIR‐triggered phototherapeutic effect of CF_5k_‐*b*PEA@siRNA NRs compared with CF_5k_‐*b*PEA NRs. Under 808 nm laser irradiation, CF_5k_‐*b*PEA@siRNA NRs were found to cause more severe cell death than CF_5k_‐*b*PEA NRs, as shown by MTS assay (Figure 4d). Further hierarchical oxidative stress response measurements corroborated that upon 808 nm laser irradiation CF_5k_‐*b*PEA@siRNA NRs could trigger higher cellular ROS level (Figure 4e) and IL‐6 cytokine release (Figure 4f), and cause more significant mitochondrial membrane depolarization (Figure 4g) and superoxide generation (Figure 4g) than CF_5k_‐*b*PEA NRs. Annexin‐V‐FITC/PI‐based apoptosis analysis by flow cytometry also supported that CF_5k_‐*b*PEA@siRNA NRs induced 10.3% of early apoptotic cells and 32.2% of late apoptotic cells while CF_5k_‐*b*PEA NRs only did 7.7% and 24.6%, respectively (Figure 4h). However, without NIR laser irradiation, all these hierarchical oxidative stress responses induced by both NRs kept the similar levels, comparable to those of untreated cells (Figure S12a–d, Supporting Information). All above results demonstrate that incorporation of DNA/Nrf2 siRNA chimera into CF_5k_‐*b*PEA NRs can further strengthen their NIR‐triggered cell injury.

Encouraged by the in vitro NIR fluorescence behaviors of various CF‐*b*PEA NRs and prominent phototherapeutic effect of CF_5k_‐*b*PEA@siRNA NRs, their in vivo fluorescence imaging and tumor growth inhibition performance were further verified in MCF‐7 tumor‐bearing mice. After intravenously administered with various CF‐*b*PEA@siRNA NRs, NIR fluorescence image of MCF‐7 tumor‐bearing mice captured at 24 h post‐administration (**Figure** **5**a) showed that free CF790 and CF_1k_‐*b*PEA@siRNA NRs did not exhibit noticeable NIR fluorescence in the whole body of mice, but CF_3.4k_‐*b*PEA@siRNA, CF_5k_‐*b*PEA@siRNA, and CF_10k_‐*b*PEA@siRNA NRs exhibited stronger fluorescence signals than free CF790. The significant tumor accumulation was clearly displayed by these NRs, where CF_5k_‐*b*PEA@siRNA NRs still showed the strongest fluorescence signal. Au element‐based ICP‐OES analysis for the tumor and major organs (Figure S13, Supporting Information) indicated that various CF‐*b*PEA@siRNA NRs had similar accumulation and circulation behaviors in mice, meaning the most promising in vivo NIR fluorescence image exhibited by CF_5k_‐*b*PEA@siRNA NRs should originate from their highest fluorescence amplification ability rather than tumor accumulation ability. Both Au and Pt elements had similar biodistribution, as demonstrated by various Au/Pt mass ratios of CF‐*b*PEA@siRNA, which are very close to their theoretical Au/Pt values of 11.60 (Table S2, Supporting Information), suggesting their excellent in vivo stability. Moreover, the photoacoustic images (Figure 5b) of tumor region further revealed the tumor accumulation behavior of CF_5k_‐*b*PEA@siRNA NRs at various time periods after the administration, demonstrating the even distribution of CF_5k_‐*b*PEA@siRNA NRs in tumor tissue. In vivo photothermal effect of CF_5k_‐*b*PEA@siRNA NRs was evaluated through infrared thermal imaging. After intravenously administered with PBS, CF_5k_‐*b*PEA NRs, or CF_5k_‐*b*PEA@siRNA NRs, the tumor region of mice were subjected to 808 nm laser irradiation (0.75 W cm^−2^, 10 min) at 24 h post‐administration. Both CF_5k_‐*b*PEA and CF_5k_‐*b*PEA@siRNA NRs could rapidly increase the tumor temperature from ≈30 to ≈45 °C within 10 min, potentially causing the similar level of cell damage (Figure 5c and Figure S14, Supporting Information).

**Figure 5:**
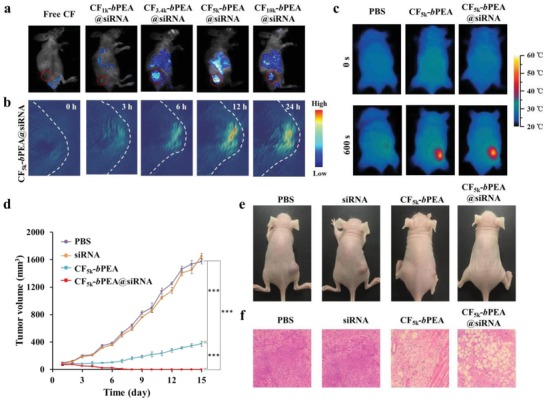
In vivo NIR laser activated theranostic property of CF_5k_‐*b*PEA@siRNA NRs on MCF‐7 tumor‐bearing mice. a) In vivo fluorescence images of mice intravenously administered with CF‐*b*PEA@siRNA NRs (equivalent to 20 mg Au per kg mice) at 24 h post‐administration. b) In vivo photoacoustic images of mice intravenously administered with CF_5k_‐*b*PEA@siRNA NRs (equivalent to 20 mg Au per kg mice) at 0–24 h post‐administration. c) Thermal infrared images of mice intravenously administered with CF_5k_‐*b*PEA or CF_5k_‐*b*PEA@siRNA NRs (equivalent to 20 mg Au per kg mouse) and irradiated by an 808 nm laser (0.75 W cm^−2^) for 10 min at 24 h post‐administration. d) Tumor growth curves of mice intravenously administered with PBS, siRNA, CF_5k_‐*b*PEA NRs, and CF_5k_‐*b*PEA@siRNA NRs (equivalent to 20 mg Au per kg mice or 15 µmol L^−1^ siRNA per kg mice) under 808 nm laser irradiation (0.75 W cm^−2^, 10 min). ***P < 0.001. e) Representative photos of mice at the end of treatment. f) H&E stained tumor sections at the end of treatment.

In vivo phototherapeutic effect of CF_5k_‐*b*PEA@siRNA NRs was further evaluated through monitoring the tumor growth rates of MCF‐7 tumor‐bearing mice. Mice were intravenously administered with PBS, siRNA, CF_5k_‐*b*PEA NRs, or CF_5k_‐*b*PEA@siRNA NRs under 808 nm laser irradiation or not. The tumor volume was measured and plotted as a function of time (Figure 5d and Figure S15a, Supporting Information). It was found that without NIR laser irradiation the tumors of mice administered with these materials could rapidly grow within 15 days, and there was no evident difference in the tumor growth rates (Figure S15a,b, Supporting Information). However, under 808 nm laser irradiation, administration with CF_5k_‐*b*PEA or CF_5k_‐*b*PEA@siRNA NRs could dramatically regress the tumor growth, where CF_5k_‐*b*PEA@siRNA NRs even completely eradicated the tumors at the end of treatment, showing more remarkable tumor regression than CF_5k_‐*b*PEA NRs (Figure 5d,e). In comparison, administration of PBS or siRNA only could weakly inhibit the tumor growth of mice. To further corroborate the therapeutic effects, the tumor tissues were dissected at the end of treatment for evaluation of the pathological changes through hematoxylin and eosin (H&E) histology analysis (Figure 5f). Without 808 nm laser irradiation, little damage was found in the tumor tissues of mice treated by these materials (Figure S15c, Supporting Information). However, under 808 nm laser irradiation, the tumor tissues of CF_5k_‐*b*PEA or CF_5k_‐*b*PEA@siRNA NRs treated mice were severely damaged, where CF_5k_‐*b*PEA@siRNA NRs were more effective than CF_5k_‐*b*PEA NRs. All these pathological results of tumor tissues are consistent with the tumor growth inhibition results, supporting CF_5k_‐*b*PEA@siRNA NRs possess more prominent phototherapeutic effect than CF_5k_‐*b*PEA NRs, which is ascribed to their attenuated antioxidant defense and hyperthermia resistance. Above results demonstrate again the combined therapeutic effect of CF_5k_‐*b*PEA@siRNA NRs based on PTT, PDT, and gene therapy.

Since the biocompatibility of the nanomaterials plays an important role for their future applications, a series of physiology parameters of mice, such as the body weight fluctuations, H&E staining of main organs, and the serum biochemistry parameters, were carefully monitored after the treatment. It could be observed that there was no noticeable difference in the body weight fluctuations of mice between treatment group and control group (Figure S16, Supporting Information). Histology analysis of major organs from mice indicated there was no appreciable abnormality or noticeable organ damage in heart, liver, spleen, lung, and kidney (Figure S17, Supporting Information). The parameters of serum biochemistry assays were also in normal range (Table S1, Supporting Information). All these in vivo biodistribution, therapeutic effects, and biocompatibility studies prove that CF_5k_‐*b*PEA@siRNA NRs hold great promise for cancer therapy.

In summary, PEA NRs could produce more potent PTT and PDT performance than PBA NRs under 808 nm laser irradiation. The NIR fluorescence intensity of CF790 could be more remarkably amplified by PEA NRs when CF790 was attached to PEA NRs through 5k PEG. Biocompatible CF_5k_‐*b*PEA NRs could be efficiently internalized into MCF‐7 cells, presenting the promising cellular NIR fluorescence and inducing severe cell damage under 808 nm laser irradiation. Further CF_5k_‐*b*PEA@siRNA NRs were prepared to knockdown Nrf2 gene in cells, capable of attenuating the cellular antioxidant defense and hyperthermia resistance capability, and inducing more significant PDT and PTT effect than CF_5k_‐*b*PEA NRs. After intravenous administration to MCF‐7 tumor‐bearing mice, CF_5k_‐*b*PEA@siRNA NRs still exhibited the most promising NIR fluorescence imaging performance and more significant tumor growth inhibition than CF_5k_‐*b*PEA NRs. Thus, CF_5k_‐*b*PEA@siRNA NRs hold great potential as a theranostic nanosystem for cancer diagnosis and therapy.

## Conflict of Interest

The authors declare no conflict of interest.

## Supporting information

Supplementary

## References

[1] a) L. Cheng , C. Wang , L. Feng , K. Yang , Z. Liu , Chem. Rev. 2014, 114, 10869; 25260098 10.1021/cr400532z

[2] a) H. Kim , K. Chung , S. Lee , D. H. Kim , H. Lee , Wiley Interdiscip. Rev.: Nanomed. Nanobiotechnol. 2016, 8, 23; 25903643 10.1002/wnan.1347

[3] a) B. Jang , J. Y. Park , C. H. Tung , I. H. Kim , Y. Choi , ACS Nano 2011, 5, 1086; 21244012 10.1021/nn102722z

[4] a) Z. Guo , S. Park , J. Yoon , I. Shin , Chem. Soc. Rev. 2014, 43, 16; 24052190 10.1039/c3cs60271k

[5] a) C. Clavero , Nat. Photonics 2014, 8, 95;

[6] a) N. S. Abadeer , M. R. Brennan , W. L. Wilson , C. J. Murphy , ACS Nano 2014, 8, 8392; 25062430 10.1021/nn502887j

[7] a) L. Gao , R. Liu , F. Gao , Y. Wang , X. Jiang , X. Gao , ACS Nano 2014, 8, 7260; 24992260 10.1021/nn502325j

[8] a) A. M. Alkilany , L. B. Thompson , S. P. Boulos , P. N. Sisco , C. J. Murphy , Adv. Drug Delivery Rev. 2012, 64, 190; 10.1016/j.addr.2011.03.005 21397647

[9] a) Z. Zheng , T. Tachikawa , T. Majima , J. Am. Chem. Soc. 2014, 136, 6870; 24779561 10.1021/ja502704n

[10] a) S. Lal , N. K. Grady , J. Kundu , C. S. Levin , J. B. Lassiter , N. J. Halas , Chem. Soc. Rev. 2008, 37, 898; 18443675 10.1039/b705969h

[11] a) S. Singh , S. Vrishni , B. K. Singh , I. Rahman , P. Kakkar , Free Radical Res. 2010, 44, 1267; 20815789 10.3109/10715762.2010.507670

[12] a) X. J. Wang , Z. Sun , N. F. Villeneuve , S. Zhang , F. Zhao , Y. Li , W. Chen , X. Yi , W. Zheng , G. T. Wondrak , P. K. Wong , D. D. Zhang , Carcinogenesis 2008, 29, 1235; 18413364 10.1093/carcin/bgn095 PMC3312612

[13] S. Son , J. Nam , J. Kim , S. Kim , W. J. Kim , ACS Nano 2014, 8, 5574. 24869928 10.1021/nn5022567

[14] a) Q. Zhang , J. Tang , L. Fu , R. Ran , Y. Liu , M. Yuan , Q. He , Biomaterials 2013, 34, 7980; 23891517 10.1016/j.biomaterials.2013.07.014

[15] X. Ye , L. Jin , H. Caglayan , J. Chen , G. Xing , C. Zheng , V. Doan‐Nguyen , Y. Kang , N. Engheta , C. R. Kagan , C. B. Murray , ACS Nano 2012, 6, 2804. 22376005 10.1021/nn300315j

[16] J. Fennell , D. He , A. M. Tanyi , A. J. Logsdail , R. L. Johnston , Z. Y. Li , S. L. Horswell , J. Am. Chem. Soc. 2013, 135, 6554. 23594230 10.1021/ja4003475 PMC3842091

[17] D. S. He , Y. Han , J. Fennell , S. L. Horswell , Z. Y. Li , Appl. Phys. Lett. 2012, 101, 113102.

[18] F.‐R. Fan , D.‐Y. Liu , Y.‐F. Wu , S. Duan , Z.‐X. Xie , Z.‐Y. Jiang , Z.‐Q. Tian , J. Am. Chem. Soc. 2008, 130, 6949. 18465860 10.1021/ja801566d

[19] X. Huang , S. Neretina , M. A. El‐Sayed , Adv. Mater. 2009, 21, 4880. 25378252 10.1002/adma.200802789

[20] J. Guo , Y. Zhang , L. Shi , Y. Zhu , M. F. Mideksa , K. Hou , W. Zhao , D. Wang , M. Zhao , X. Zhang , J. Lv , J. Zhang , X. Wang , Z. Tang , J. Am. Chem. Soc. 2017, 139, 17964. 29155572 10.1021/jacs.7b08903

[21] a) T. L. Doane , Y. Cheng , A. Babar , R. J. Hill , C. Burda , J. Am. Chem. Soc. 2010, 132, 15624; 20958038 10.1021/ja1049093

[22] X. Xia , M. Yang , Y. Wang , Y. Zheng , Q. Li , J. Chen , Y. Xia , ACS Nano 2012, 6, 512. 22148912 10.1021/nn2038516 PMC3265621

[23] a) M. C. Jaramillo , D. D. Zhang , Genes Dev. 2013, 27, 2179; 24142871 10.1101/gad.225680.113 PMC3814639

