# Supplementary material for: Anisotropic Plasmonic Metal Heterostructures as Theranostic Nanosystems for Near Infrared Light‐Activated Fluorescence Amplification and Phototherapy
Source: Adv Sci (Weinh). 2019 Apr 5;6(11):1900158. doi: 10.1002/advs.201900158 (PMC6548947; doi:10.1002/advs.201900158)
Supplement: Supplementary file 1 — Supplementary [file ADVS-6-1900158-s001.pdf]

Copyright WILEY-VCH Verlag GmbH & Co. KGaA, 69469 Weinheim, Germany, 2019.

Supporting Information

for *Adv. Sci.,* DOI: 10.1002/advs.201900158

Anisotropic Plasmonic Metal Heterostructures as Theranostic Nanosystems for Near Infrared Light-Activated Fluorescence Amplification and Phototherapy

*Yun Chang, Yanlin Feng, Yan Cheng, Runxiao Zheng, Xiaqing Wu, Hui Jian, Dawei Zhang, Zhaohui Tang, Zhenxin Wang, Jiaming Hao, and Haiyuan Zhang**

Copyright WILEY-VCH Verlag GmbH & Co. KGaA, 69469 Weinheim, Germany, 2019.

Supporting Information

Anisotropic Plasmonic Metal Heterostructures as Theranostic Nanosystems for Near Infrared Light-Activated Fluorescence Amplification and Phototherapy

Yun Chang, Yanlin Feng, Yan Cheng, Runxiao Zheng, Xiaqing Wu, Hui Jian, Dawei Zhang, Zhaohui Tang, Zhenxin Wang, Jiaming Hao, and Haiyuan Zhang *

Experimental Procedures

Chemicals. All chemicals were used as received without further purification or modification unless otherwise indicated. Ultra-pure water used in all experimental procedures was obtained from a Milli-Q water purification system (Millipore, Bedford, USA).

Preparation of Au NRs. Au NRs were synthesized by a seed-mediated method. Firstly, for the seed solution preparation, 5 mL of hexadecyl trimethyl ammonium bromide (CTAB) aqueous solution (0.2 mol L^{-1}) was mixed with 5 mL of gold chloride hydrate ($HAuCl_4$) aqueous solution (0.5 mmol L^{-1}) under vigorous stirring for 2 min, and 0.6 mL of ice-cold sodium borohydride ($NaBH_4$) aqueous solution (0.01 mmol L^{-1}) was added to the mixture under vigorous stirring for another 2 min. Then, the seed solution was kept at 30 ^{o}C for 2 h before use. Secondly, for Au NR growth process, 3.6 g of CTAB and 0.44 g of 5-bromosalicylic acid (5-BrSA) were dissolved in 100 mL of warm water (55 ºC). Then, 1.92 mL of 4 mmol L^{-1} silver nitrate ($AgNO_3$) solution was added, and the mixture was kept undisputed at room temperature for 15 min. Then, 100 mL of 1.0 mmol L^{-1} $HAuCl_4$ aqueous solution was added to the mixture. After 15 min of a gentle stirring process, 0.512 mL of 0.1 mol L^{-1} ascorbic acid (AA) aqueous solution was added to this mixture with vigorous stirring until the mixture became colorless. Finally, 0.32 mL of seed solution was added to the entire growth solution, and the mixture was stirred for 30 s and left undisturbed at 30 ºC for 12 h. The resultant solution was centrifuged at 7,500 rpm for 10 min and washed with water for three times, and achieved Au NRs were resuspended in 50 mL of water for further use.

Preparation of PEA and PBA NRs. For preparation of PEA NRs, 10 mL of above Au NR aqueous suspension was added to 10 mL of an aqueous solution containing 0.184 g of CTAB and 0.022 g of 5-BrSA. After continuous gentle stirring, 78.5 μL of 10 mmol L^{-1} chloroplatinic acid (H_2PtCl_4) aqueous solution was added and the resulting mixture was kept

undisturbed for 30 min at 40 ^{o}C. 157 μL of 0.1 mol L^{-1} AA aqueous solution was added to the above mixture, followed by 30 s of stirring. The resulting solution was kept undisturbed at 40 ^{o}C for 12 h, and PEA NRs were collected through centrifugation at 7,000 rpm for 10 min and washed with water for three times.

For preparation of PBA NRs, a similar procedure was performed as described above but without the addition of 5-BrSA.

Preparation of CF-PEA NRs using various molecular weights of PEG as linkers. CF790 was first labeled on different molecular weights of PEG (Mw=1, 3,4, 5, or 10 kDa). Briefly, 1 mg of NH_2-$PEG_{1\sim10k}$-SH (Seebio, Shanghai, China) was dissolved in 0.5 mL of 0.1 mol L^{-1} sodium bicarbonate buffer (pH=8.3), followed by addition with 5 μL of 10 mmol L^{-1} NHS modified CF790 (Biotium, Inc., USA). The resulting solution was kept at room temperature for 1 h under shaking, and loaded onto a G-75 column for purification of CF790 labeled PEG-SH (CF-$PEG_{1\sim10k}$-SH). In a typical process of CF-$PEG_{1\sim10k}$-SH conjugation to PEA NRs, 0.5 mL of CF-$PEG_{1\sim10k}$-SH solution (2 mg mL^{-1}) was added to 2.5 mL of PEA NR aqueous suspension (0.5 mg mL^{-1}), followed by addition of 2 mL of water. The reaction mixture was vortexed immediately and then incubated at 4 ^{o}C for overnight, followed by centrifugation at 8,000 rpm for 5 min to remove free CF-$PEG_{1\sim10k}$-SH. The resulting CF-PEA NRs were resuspended in 5 mL of water.

In order to quantify the amount of CF790 on CF-PEA NRs, 2.5 mL of 40 mmol L^{-1} potassium cyanide (KCN) aqueous solution and 2 mL of water were added to 500 μL of above CF-PEA NR suspension. After 30 min of incubation at room temperature, the fluorescent spectra (λ_{ex}≈784 nm, λ_{em}≈806 nm) of the resulting solution were collected, and the concentration of CF790 in each sample was determined based on the calibration curve of free CF790 aqueous solution.

Preparation of CF-*b*PEA NRs. 5 mL of 2 mg mL^{-1} CF-PEA NR suspension was mixed with 5 mL of aqueous solution containing 0.01 mg mL^{-1} SH-$PEG_{3.4k}$-CPP (SH-$PEG_{3.4k}$-AGYLLGHINLHHLAHL(Aib)HHIL-NH_2, ChinaPeptide, Shanghai, China) under stirring, and the resultant solution was kept at 4 ^oC under dark condition and N_2 atmosphere for 48 h under constant stirring. The resulting CF-*b*PEA NRs were collected through centrifugation and washed with water for three times.

Preparation of CF_{5k}-*b*PEA@siRNA NRs. Prior to modification, i-motif DNA (5' to 3' , SH-AAAAAAAAAAAAAACCCCAA, TaKaRa, Inc., China) was hybridized with DNA linker /$siRNA_{Nrf2}$ complex (sense sequences, 5' to 3' , UAAUUGUCAACUUCU GUCA(dTdT)TTGGGTTATTTTTT, and anti-sense sequence, 5' to 3' , UGACAGAAG UUGACAAUUA(dTdT), TaKaRa, Inc., China) to form i-motif DNA/siRNA $_{Nrf2}$ chimera. 10 μL of 10 μmol L^{-1} DNA linker/$siRNA_{Nrf2}$ complex and 10 μL of 10 μmol L^{-1} i-motif DNA was mixed, and the mixture was added to 500 μL of buffer solution (containing 300 mmol L^{-1} NaCl, 25 mmol L^{-1} Tris acetate, pH=8.2). Then, this mixture was incubated at 4 ^oC for 12 h under shaking, followed by addition of 10 μL of 1 mg mL^{-1} Tris(2-carboxyethyl)phosphine hydrochloride (TCEP) for activation of the disulfide functionality at the end of thiolated i-motif DNA. Then, 100 μL of the activated DNA/$siRNA_{Nrf2}$ chimera solution was added to 3 mL of CF_{5k}-*b*PEA NR aqueous solution (~50 μg mL^{-1}) with gentle shaking. After 16 h of incubation at 4 ^oC, 30 μL of 500 mmol L^{-1} Tris acetate (pH=8.2) buffer was added dropwise to the mixture, followed by addition of 300 μL of 1.0 mol L^{-1} sodium chloride (NaCl) aqueous solution. Then, the mixture was incubated for 12 h. The resultant solution was centrifuged at 8,000 rpm for 5 min, and then CF_{5k}-*b*PEA@siRNA NRs were resuspended in 3 mL of buffer (containing 300 mmol L^{-1} NaCl, 25 mmol L^{-1} Tris acetate, pH=8.2) for further applications.

FDTD simulation. The electric field distribution was calculated using Lumerical Solutions, a commercially available FDTD simulation software package, with perfectly matched layers boundary conditions. The optical constants of Au were adopted from tabulated value for bulk gold measured by Johnson and Christy.[24] The size of the nanorod was taken to match the average value. Specifically, Au NR was modeled as a cylinder capped two half-spheres at the ends. For the model of PEA NR, ellipse Pt spheres were located at both ends of the Au NR. For the model of the PBA NR, a thin layer Pt was homogeneously located on the surface of the Au NR. For NRs dispersed in the aqueous solution, the refractive index of the medium was set to be 1.33.

Characterization. TEM images were taken using a JEOL microscope (1200EX II) operated at 120 kV. UV-visible spectra were recorded with a Cary V550 spectrometer. Zeta potential and hydrodynamic size were collected on Malvern Nanosizer ZS (Malvern Panalytical, Australia). The NIR fluorescence spectroscopy measurement was carried out on a Fluoromax-3 spectrofluorimeter (Horiba Scientific, Japan).

Transient absorption spectroscopy measurement. Non-degenerate pump-probe experiments were performed with 100-fs pulse from a 1 kHz Ti:sapphire laser amplifier (Libra) and an optical parametric amplifier (OPerA Solo). Pump pulse from the OPerA Solo doubled in a β-barium borate crystal to provide a tunable wavelength source. Probes pules were taken directly from the Libra. Time-delay between the pules was controlled by a motion control system with a 4-ns time range. Focused beam spot sizes were 400 μm for the pump and 240 μm for the probe. All samples (optical densities were similar at pump wavelength) were dispersed in water and subsequently sealed in a transparent cell during measurements, which was mounted on a rotating stage for keeping the pump-excited fraction of sample fresh. All measurements were taken under ambient conditions.

Photothermal performance measurement. In order to measure the photothermal conversion performance of Au, PEA, and PBA NRs, 0.6 mL of an aqueous suspension containing 50 μg mL^{-1} NRs (equivalent to Au content) was put into a quartz cuvette, followed by exposure to an 808 nm laser at 0.75 W cm^{-2} for different time periods. During the experimental process, a thermocouple probe with accuracy of ±0.1 °C was inserted into the suspension perpendicular to the laser path for avoiding direct light irradiation on the probe, and the temperature of the suspension was recorded every 20 seconds until 10 min.

ESR measurement. $^1O_2$ measurement. To 100 μL of 25 μg mL^{-1} NRs D_2O suspension was added 40 μL of 1 mol L^{-1} TEMP aqueous. The resulting solution was mixed well and exposed to an 808 nm laser (0.75 W cm^{-2}) for 10 min.

$O_2^{\bullet-}$ measurement. To 50 μL of 25 μg mL^{-1} NRs suspension in dimethyl sulfoxide (DMSO) was added 10 μL of 1 mol L^{-1} DMPO in DMSO. The resulting solution was mixed well and exposed to an 808 nm laser (0.75 W cm^{-2}) for 10 min.

•OH measurement. To 100 μL of 25 μg mL^{-1} NRs H_2O suspension was added 15 μL of 1 mol L^{-1} DMPO aqueous. The resulting solution was mixed well and exposed to an 808 nm laser (0.75 W cm^{-2}) for 10 min, and immediately subjected to ESR measurement by Bruker A300 EPR electron paramagnetic resonance spectrometer. Parameter setting: microwave power, 10.12 Mw; frequency, 9.8 GHz; time constant, 40.96 ms; scan width, 100 G.

Cell culture. Human breast cancer cells (MCF-7) were cultured in vented T-75 cm^2 flasks at 37 °C in a humidified 5% CO_2 atmosphere and passaged at 70-80% confluency every 2-4 days. MCF-7 cells were cultured in Dulbecco's Modified Eagle's Medium (DMEM) (containing 10% fetal bovine serum, 100 units mL^{-1} penicillin, and 100 units mL^{-1} streptomycin). Acidic culture medium (pH=6.5) was prepared by the addition of lactate to DMEM culture medium.[25]

The fluorescence and dark-field images of cells treated with CF-*b*PEA NRs. 1×10^5 cells in 400 μL of culture medium were plated in each well of an 8-well chamber slide for overnight growth. After the removal of the culture medium, cells were incubated with 400 μL of 25 μg mL^{-1} various CF-*b*PEA NRs (equivalent to Au) suspended in acidic culture medium at 37 ^oC for 6 h. Then, the medium was replaced with 400 μL of normal DMEM culture medium for additional 6 h of incubation. Cells were washed three times with PBS and fixed with 400 μL of 4% paraformaldehyde PBS solution for 2 h at room temperature. After washing with PBS three times, the cell nuclei were stained by 1 μmol L^{-1} Hoechst 33342 for 30 min. The NIR fluorescent images were captured under a confocal laser scanning microscopy (ZEISS LSM780, Germany) with an excitation wavelength of 633 nm and an emission wavelength of 670-810 nm. The dark field images were captured with a Leica DM 6000 upright microscope (Leica Microsystems Inc., Germany) using Xenon illumination along with dark-field objective and a Retiga Exi camera with 12-bit ultra-sensitive CCD camera detector.

***In vitro* cytotoxicity measurement**. Cell viability was determined by an MTS assay. 1×10^4 MCF-7 cells in 100 μL of culture medium were plated in each well of a 96-mutiwell plate for overnight growth. For the treatment without NIR laser irradiation, the culture medium was removed and cells were treated with 100 μL of acidic culture medium containing 6.3, 12.5, 25, 50, and 100 μg mL^{-1} CF_{5k}-*b*Au, CF_{5k}-*b*PEA, or CF_{5k}-*b*PBA NRs (equivalent to Au content) for 6 h. Then, the acidic culture medium was removed, and 100 μL of normal DMEM culture medium was added for further 18 h of incubation. For the treatment with NIR laser irradiation, the culture medium was removed and cells were treated with 100 μL of acidic culture medium containing different concentrations of above various NRs for 6 h, followed by 5 min of 808 nm laser irradiation at a power density of 0.5 W cm^{-2}. Then, the acidic culture medium was removed and 100 μL of normal DMEM culture medium was

added for further 18 h incubation. After above treatment with or without NIR laser irradiation, the culture medium was removed and 100 μL of culture medium containing 16.7% of MTS stock solution was added into each well. After 4 h of treatment at 37 ^{o}C in a humidified 5% CO_2 incubator, the plate was centrifuged at 4,000 rpm for 10 min to spin down the cell debris in Xiangyi L535R with a microplate rotor. 80 μL of suspension was transferred into a new 96-well plate and the absorbance of formazan was read at 490 nm on SpectraMax M5 microplate reader.

Live/dead cell staining assay. 1×10^5 MCF-7 cells in 400 μL of culture medium were plated in each well of an 8-well chamber slide for overnight growth. For the treatment without NIR laser irradiation, the culture medium was removed and cells were treated with 400 μL of acidic culture medium containing 25 μg mL^{-1} CF$_{5k}$-*b*Au, CF$_{5k}$-*b*PEA, or CF$_{5k}$-*b*PBA NR suspension (equivalent to Au content) for 6 h. Then, the cell medium was removed, and 400 μL of normal DMEM culture medium was added for further 18 h incubation. For the treatment with NIR laser irradiation, cells were similarly treated with 400 μL of acidic culture medium containing 25 μg mL^{-1} NR suspensions for 6 h, followed by 5 min of 808 nm laser irradiation at a power density of 0.5 W cm^{-2}. Then, the acidic culture medium was replaced with 400 μL of normal DMEM culture medium for additional 18 h incubation. After above treatment with or without NIR laser irradiation, cells were stained by 400 μL of culture medium containing 1 μmol L^{-1} Calcein-AM (KeyGEN BioTECH, China) and 1 μmol L^{-1} propidium iodide (KeyGEN BioTECH, China) for 30 min. After washing with PBS three times, cells were visualized using Olympus BX-51 optical system microscope (Tokyo, Japan).

Agarose gel electrophoresis to analyze the siRNA release from CF$_{5k}$-*b*PEA@siRNA NRs. The pH-dependent siRNA release behavior of 50 μg mL^{-1} CF$_{5k}$-*b*PEA@siRNA NRs (equivalent to Au content) was investigated in a buffer solution containing 300 mmol L^{-1}

NaCl and 25 mmol L^{-1} Tris acetate with a pH of 3.0, 4.5, 5.5, 6.5, 7.4, 8.0, or 9.0, respectively. After 2 h of incubation, 25 μL of the mixture was mixed with 5 μL of loading buffer and loaded onto each well of 4.0 wt% agarose gel containing 0.5 μg mL^{-1} ethidium bromide. Gel was run at 100 V for 1 h and subsequently imaged using a Tanon 2500R Gel Imaging System (Tanon Corp., Shanghai, China).

Confocal laser scanning microscopy to analyze the intracellular trafficking of CF_{5k}-*b*PEA@FITC-siRNA NRs with or without CPP. 1×10^5 MCF-7 cells in 400 μL of culture medium were plated in each well of an 8-well chamber slide for overnight growth. 400 μL of CF_{5k}-*b*PEA@FITC-siRNA NRs with or without CPP in acidic culture medium was incubated with MCF-7 cells for 6 h. After the treatment, cells were washed with PBS three times and stained by 400 μL of 2 μmol L^{-1} Lyso-tracker Red (KeyGEN BioTECH, China) for 30 min. Cell nuclei were stained by 400 μL of 1 μmol L^{-1} Hoechst 33342 for 30 min. After the staining, cells were washed with PBS three times and fixed by 400 μL of 4% paraformaldehyde solution in PBS for 2 h at room temperature. The fluorescent images were captured under a confocal laser scanning microscopy (ZEISS LSM780, Germany).

Western blotting analysis of Nrf2, HO-1, and HSP 70 proteins. 1.6×10^5 MCF-7 cells in 1.6 mL of DMEM were seeded in each well of six-well plate for overnight growth. Cells were treated with 1.6 mL of 25 μg mL^{-1} CF_{5k}-*b*PEA@siRNA or CF_{5k}-*b*PEA NRs (equivalent to Au content) in acidic culture medium for 6 h. Then, the cell medium was removed, and 1.6 mL of normal DMEM culture medium was added for another 6 h of incubation. After the treatment, cells were washed with PBS three times and collected by scraping, and lysed by a lysis buffer containing Triton-100 and Protease inhibitors. After the protein content was determined by a Bradford method, 30 μg of protein from each sample was electrophoresed by 10% SDS-PAGE and transferred to a PVDF membrane (Millipore Corp., USA). After blocking, the membranes were incubated with anti-Nrf2 monoclonal antibody (1:800; ENZO

Life Sciences, USA) or anti-HO-1 monoclonal antibody (1:1,000; ENZO Life Sciences, USA) or HSP 70 monoclonal antibody (1:1,000; Beyotime, China) for 2 h, followed by 1 h of incubation with secondary antibody before the addition of HRP-conjugated SuperSignal West Pico chemiluminescent substrate (Thermo Fisher Scientific, Waltham, MA, USA), and the protein was detected by chemiluminescence imaging system (Tanon 5200 Multi, Tanon, China).

Flow cytometric analysis to determine the intracellular ROS level. The intracellular ROS was measured by flow cytometry based on H_2DCFDA assay. 1.6 $\times 10^5$ MCF-7 cells were seeded in each well of six-well plates for overnight growth. For the treatment without NIR laser irradiation, cells were treated with 1.6 mL of 10 μg mL^{-1} CF_{5k}-*b*PEA or CF_{5k}-*b*PEA@siRNA NRs (equivalent to Au content) in acidic culture medium for 6 h. Then, the cell medium was removed, and 1.6 mL of normal DMEM culture medium was added for another 6 h of incubation. For the treatment with NIR laser irradiation, cells were similarly treated with NRs in acidic culture medium for 6 h, followed by 5 min of 808 nm laser irradiation (0.5 W cm^{-2}). Then, the cell medium was then removed, and 1.6 mL of normal DMEM culture medium was added for another 6 h of incubation. After each treatment, cells were washed with PBS three times, and incubated with 10 μmol L^{-1} H_2DCFDA at 37 ºC for 30 min. Then, cells were trypsinized and washed with PBS for three times, and analyzed using a FACS-Calibur flow cytometer (BD Biosciences).

Fluorescence microscopy analysis of mitochondrial dysfunction. Mitochondrial membrane depolarization and superoxide generation were detected by JC-1 and Mitosox Red fluorescence indicators (Thermo Fisher Scientific, Waltham, MA, USA), respectively. 1×10^5 MCF-7 cells in 400 μL of culture medium were plated in each well of an 8-well chamber slide for overnight growth. For the treatment without NIR laser irradiation, cells were treated with 400 μL of 10 μg mL^{-1} CF_{5k}-*b*PEA or CF_{5k}-*b*PEA@siRNA NRs (equivalent to Au

content) in acidic culture medium for 6 h. Then, the cell medium was removed, and 400 μL of normal DMEM culture medium was added for another 6 h of incubation. For the treatment with NIR laser irradiation, cells were similarly treated with NRs in acidic culture medium for 6 h, followed by 5 min of 808 nm laser irradiation (0.5 W cm^{-2}). Then, the cell medium was removed, and 400 μL of normal DMEM culture medium was added for another 6 h of incubation. After each treatment, cells were washed with PBS three times and stained with 5 μmol L^{-1} JC-1 or 5 μmol L^{-1} Mitosox Red for 30 min. Cell nuclei were stained with 1 μmol L^{-1} Hoechst 33342 for 30 min. After staining, cells were washed with PBS three times and fixed by 400 μL of 4% paraformaldehyde solution in PBS for 2 h at room temperature. The fluorescence images were performed on Olympus BX-51 optical system microscope (Tokyo, Japan) with 20×objective.

Flow cytometric analysis of cell apoptosis. 8 $\times 10^4$ MCF-7 cells were seeded in each well of 12-well plates for overnight growth. For the treatment without NIR laser irradiation, cells were treated with 800 μL of 10 μg mL^{-1} CF_{5k}-*b*PEA or CF_{5k}-*b*PEA@siRNA NRs (equivalent to Au content) in acidic culture medium for 6 h. Then, the cell medium was then removed, and 800 μL of normal DMEM culture medium was added for another 6 h of incubation. For the treatment with NIR laser irradiation, cells were similarly treated with NRs in acidic culture medium for 6 h, followed by 5 min of 808 nm laser irradiation (0.5 W cm^{-2}). Then, the culture medium was removed, and 800 μL of normal DMEM culture medium was added for another 6 h of incubation. After each treatment, cells were detached with trypsin (without ethylenediaminetetraacetic acid (EDTA)), washed twice with PBS, then cells were treated with Annexin V-FITC apoptotic analysis kit (KeyGEN BioTECH, China). Then the cell apoptosis was analyzed by FACS-Calibur flow cytometer (BD Biosciences).

Animals. Female BALB/c athymic nude mice were obtained from Beijing Vital River Experiment Animal Technology Co. Ltd. with body weight around 20 g. The mice were kept

in the standard conditions (around 20 ^{o}C room temperature, normal humidity) and housed in stainless steel cages. Distilled water and sterilized food were available. All animal studies were performed in Center for Experiment Animals, Jilin University, and all the procedure were compliant with animal ethics committee of Jilin University.

***In vivo* imaging and biodistribution analysis**. Nude mice were subcutaneously inoculated with 100 μL of PBS containing 5×10^6 MCF-7 cells at the back. When the tumor reached to 100 mm^3, mice were intravenously administered with 100 μL of CF-*b*PEA@siRNA NRs (equivalent to 20 mg Au/kg mice). Then, the mice were anesthetized at 24 h post-injection and placed on nonfluorescent black plates for whole body fluorescence imaging using a Maestro *in vivo* fluorescence imaging system (Cambridge Research & Instrumentation, Inc., Woburn, MA). Spectral fluorescent images were obtained for the purpose of determining the target-to-background ratios of tumor implants versus normal anatomy. At the end of treatment, mice were sacrificed and the major organ tissues (heart, liver, spleen, lung, kidney, and tumor) were collected and placed into 10 mL of concentrated HNO_3 for overnight pre-digestion. Then, 3 mL of 30% H_2O_2 was added and heated to boiling temperature for 1 h until digestion was completed. After the solution cooled to room temperature, the solution in each flask was diluted to 5 mL with 2% HNO_3. The obtained liquid was subjected to ICP-OES (Thermo Scientific ICAP6300) analysis based on Au element.

***In vivo* photoacoustic imaging**. Photoacoustic imaging was performed on the MCF-7 tumor xenograft mouse model by using the MOST system and processing software. For *in vivo* photoacoustic imaging, the tumor-bearing nude mice were intravenously administered with 100 μL of CF_{5k}-*b*PEA@siRNA NRs (equivalent to 20 mg Au/kg mice). Images were captured at 0, 3, 6, 12, and 24 h post-injection. The mice were anesthetized with isoflurane, and placed into a water bath to maintain their body temperature at 37 °C for following tumor imaging.

***In vivo* therapeutic evaluation**. For *in vivo* therapeutic evaluation of CF_{5k}-*b*PEA@siRNA NRs, 100 μL of PBS containing 1×10^6 MCF-7 cells was subcutaneously injected at the back of mice. When the tumor sizes were approaching approximately 100 mm^3, the mice were divided into eight groups: (a) PBS, (b) siRNA, (c) CF_{5k}-*b*PEA NRs, (d) CF_{5k}-*b*PEA@siRNA NRs, (e) PBS+NIR laser, (f) siRNA+NIR laser, (g) CF_{5k}-*b*PEA NRs+NIR laser, (h) CF_{5k}-*b*PEA@siRNA NRs +NIR laser. Mice were intravenously administered with 100 μL of PBS, siRNA, CF_{5k}-*b*PEA NRs, or CF_{5k}-*b*PEA@siRNA NRs (equivalent to 20 mg Au/kg mice or 15 μmol L^{-1} siRNA/kg mice). For NIR irradiation group, at 24 h post-injection, the tumor region were irradiated with an 808 nm laser at the power intensity of 0.75 W cm^{-2} for 10 min. Temperature changes in the tumor region was recorded by infrared camera (FLIR, USA) during NIR irradiation. Tumor growth and mouse weigh were measured in the following days. The tumor size was defined as $V=ab^2/2$, where a and b are the tumor length and width, respectively. For histology analysis, the major organs (heart, liver, spleen, lung, kidney, and tumor) were harvested from mice in each group at the end of treatments, and fixed in 10% neutral buffered formalin, and embedded in paraffin. Slices with a thickness of 5 μm were incised and stained with hematoxylin and eosin (H&E). Pathology was examined by a digital microscope.

Serum biochemistry measurement. There were four groups of mice for the sub-chronic toxicity test, (a) health mice intravenously administered with PBS, (b) health mice intravenously administered with CF_{5k}-*b*PEA@siRNA NRs (equivalent to 20 mg Au/kg mice), (c) tumor-bearing mice intravenously administered with PBS, and (d) tumor-bearing mice intravenously administered with CF_{5k}-*b*PEA@siRNA NRs (equivalent to 20 mg Au/kg mice) and irradiated by 808 nm laser (0.75 W cm^{-2}, 10 min). The serum biochemistry was detected at 20 days post-treatment. Blood samples (approximately 0.5 mL $sample^{-1}$) were collected via orbital puncture from each mouse. The serum biochemistry parameters including alanine

aminotransferase (ALT), aspartate aminotransferase (AST), blood urea nitrogen (BUN), albumin (ALB), and creatinine (CER), were measured by Hitachi 7020 automatic biochemical analyzer.

Statistical analysis. All data were presented as mean or mean±standard deviation. Statistical significance was evaluated using two-tailed heteroscedastic *Student's t*-tests according to the TTEST function in Microsoft Excel.

Table S1. Serum biochemistry data including liver function markers, blood urea nitrogen (BUN) levels, and creatinine (CRE). Healthy female Balb/c mice intravenously administered with CF_{5k}-*b*PEA@siRNA (20 mg Au/kg mouse) or PBS (control) were sacrificed at 20 day post-administration for blood collection. MCF-7 tumor-bearing female Balb/c mice intravenously administered with PBS without laser irradiation or CF_{5k}-*b*PEA@siRNA (20 mg Au/kg mouse) with 808 nm laser irradiation (0.75 W cm^{-1}, 10 min) were sacrificed after 15 days of treatment for blood collection. Reference ranges of hematology data of healthy female Balb/c mice were obtained from Charles River Laboratories (http://www.criver.com).

	Reference range	Health mice		Tumor-bearing mice	
		**PBS**	**CF_{5k}-*b*PEA @siRNA**	**PBS**	**CF_{5k}-*b*PEA @siRNA**
ALT (IU)	105±65	139.2±45.57	159.7±31.8	175.44±34.93	171.4±34.1
AST (IU)	217±150	242.48±24.2	272.19±22.68	299±24.91	292.1±24.3
ALB (IU)	187±78	218.7±46.43	252.69±19.84	277.58±21.8	271.2±21.3
BUN (mM)	20±13	25.93±10.3	32.8±13.03	30.03±13.9	25.3±13.9
CRE (mM)	35±15	37.19±6.82	47.04±8.62	51.67±9.47	50.5±9.3

Table S2. Mass ratios of Au/Pt in various organs of MCF-7 tumor-bearing mice at 24 h post-administration with CF-*b*PEA@siRNA NRs based on Au and Pt elements analyzed by ICP-OES.

Mass Ratio of Au/Pt	Tumor	Heart	Liver	Spleen	Lung	Kidney
CF_{1k}-*b*PEA@siRNA	11.30	12.13	11.44	11.41	11.55	11.45
$CF_{3.4k}$-*b*PEA@siRNA	11.34	11.89	11.09	11.99	11.92	11.27
CF_{5k}-*b*PEA@siRNA	11.82	11.02	11.42	11.42	12.30	11.33
$CF10_{k}$-*b*PEA@siRNA	11.84	11.37	11.22	12.21	12.13	11.10

The mass ratios of Au/Pt in various organs of MCF-7 tumor bearing mice are very close to their theoretical ratio of 11.60 in CF-*b*PEA@siRNA NRs, suggesting the excellent *in vivo* stability of metal heterostrucutres.

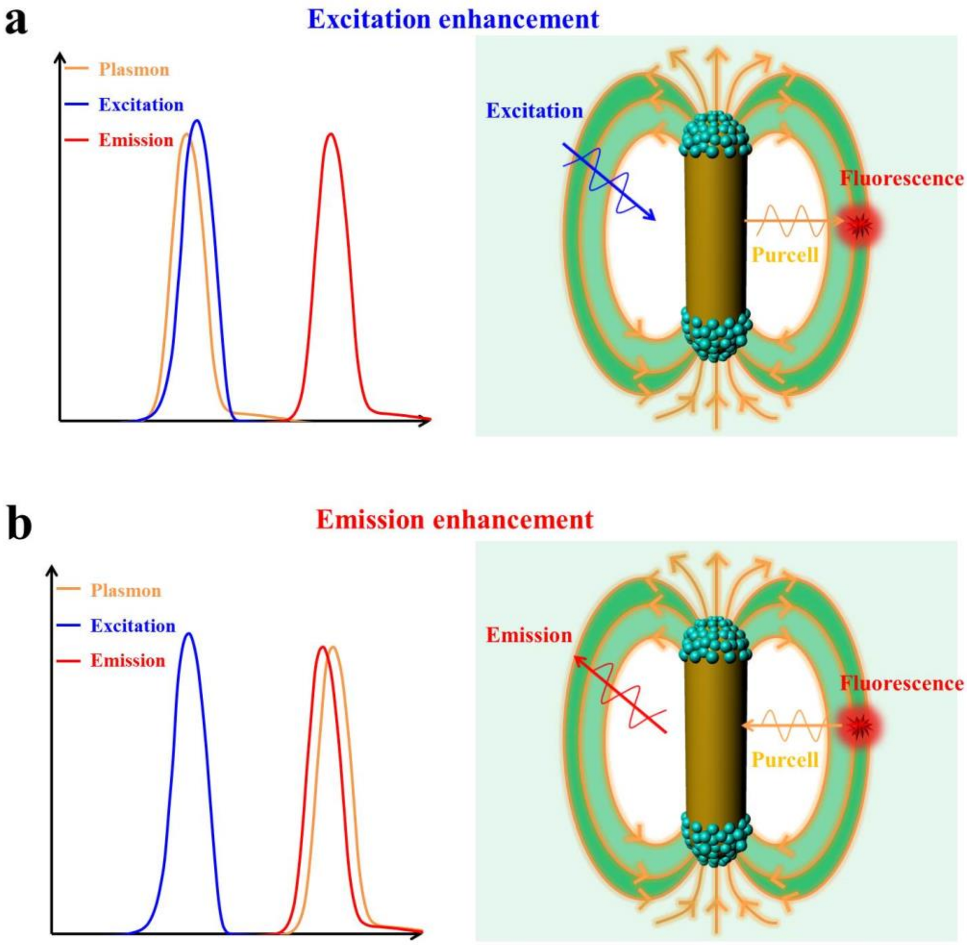

Scheme S1. Purcell effect-dominated fluroescence enhancment. (a) If the plasmon overlaps with the absorption of the fluorophore, the excitation rate of the fluorophore will be enhanced. (b) If the plasmon overlaps with the emission of the fluorophore, the emission rate of the fluorophore will be enhanced.

The spectral overlap between the plasmon and the fluorophore could effectively lead to an enhancement of the fluorescence intensity, which is dominated by Purcell effect.[1] The Purcell effect can be understood as follow. If a radiative dipole is placed in the plasmonic resonant cavity, the fluorescence emission intensity will be amplified on-resonance.[2] This is because the plasmonic resonance cavity modifies the local density of optical states (LDOS).[3] The plasmonic electromagnetic field can effectively enhance the LDOS and induce a strong Prucell enhancement effect,[4] as a result of an increase of the radiative rate of the fluorophore.[5] If the fluorophore's absorption overlaps with the plasmon (Scheme S2a), the excitation rate of the fluorophore will be enhanced. If the fluorophore's emission overlaps with the plasmon (Scheme S2b), the emission rate of the fluorophore will be enhanced.

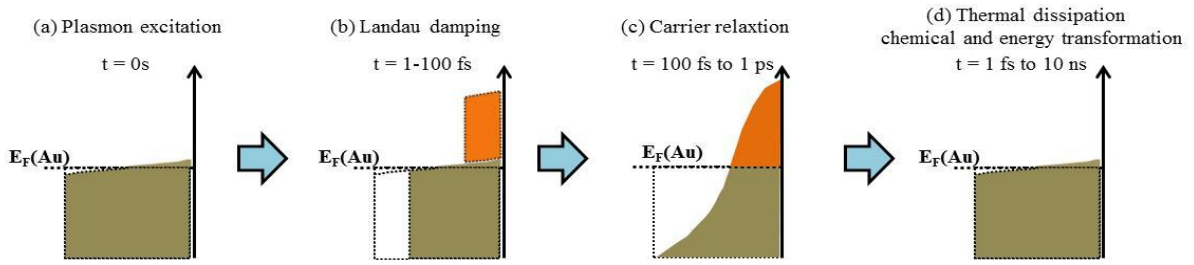

Scheme S2. Photoexcitation and subsequent relaxation processes following the illumination of Au nanoparticles with a laser pulse, and characteristic timescales. (a) Initially, the electrons follow a Fermi-Dirac distribution at the thermal temperature of the system. (b) The photoexcitation of electrons from filled states to unfilled states generates a non-thermal electron distribution. (c) The energetic hot electrons will redistribute their energy on a timescale ranging from 100 fs to 1 ps. (d) Finally, the energetic hot electrons transferred to the surrounding environment through both thermal dissipation and chemical and energy transformation processes.

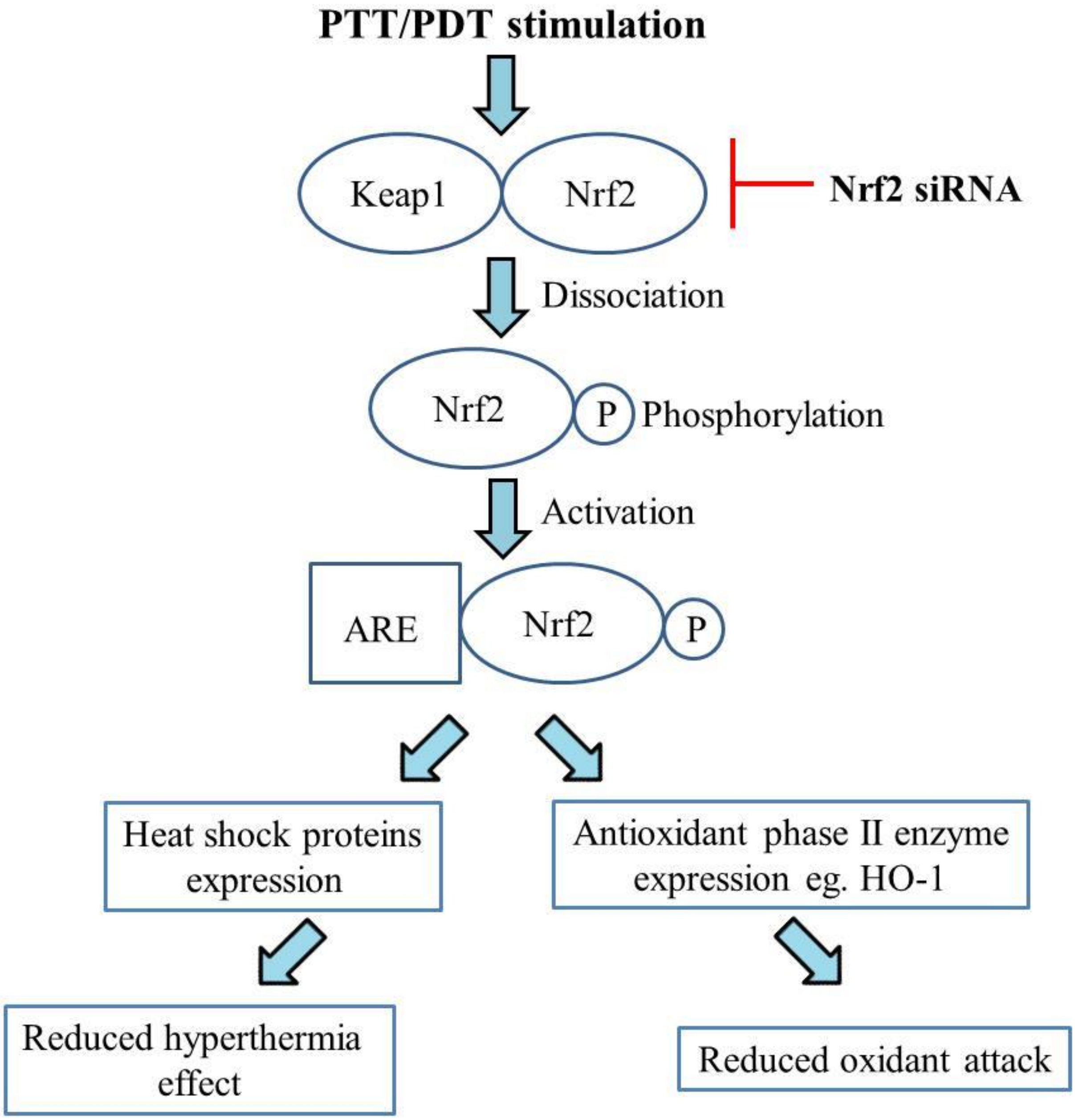

Scheme S3. In cells, PDT and PTT can usually trigger phase II enzyme and heat shock protein expression to reduce the oxidant attacking and hyperthermia effect.

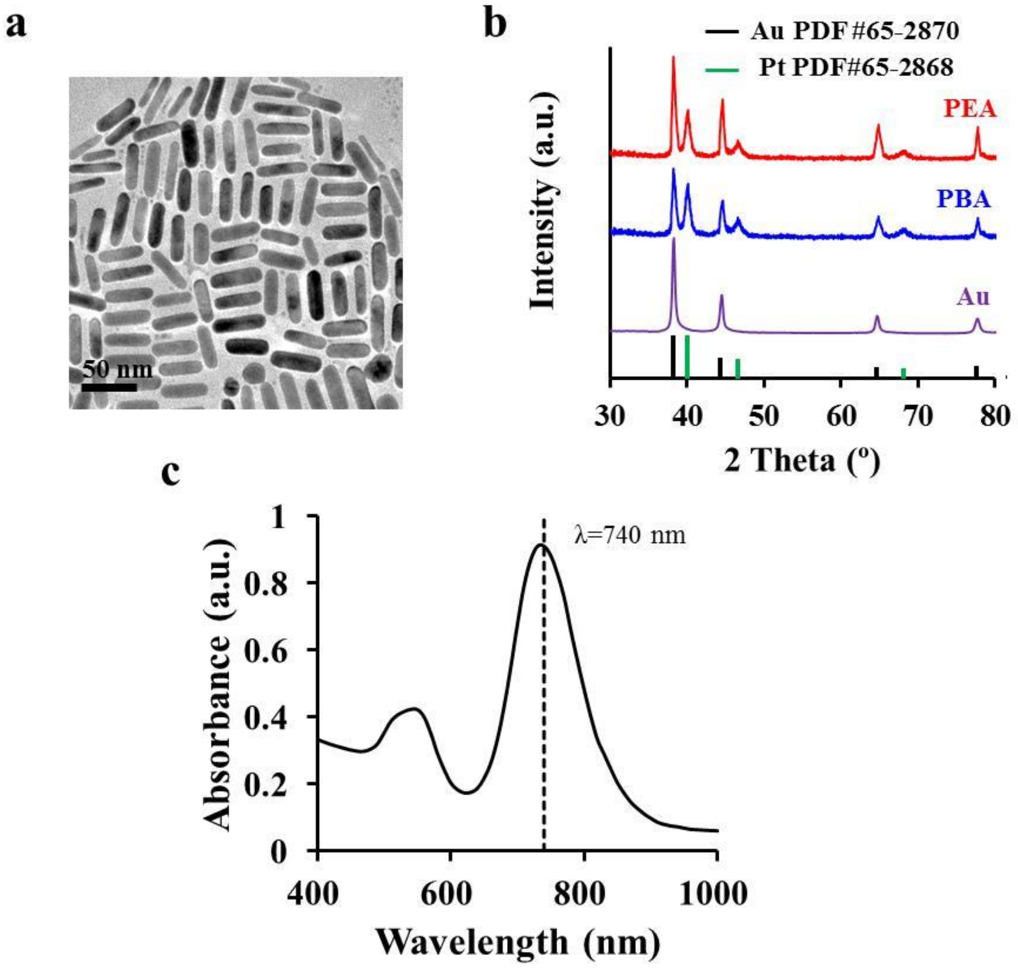

Figure S1. Characterization of Au NRs. (a) TEM images. (b) XRD pattern. (c) UV-Vis-NIR absorption spectra.

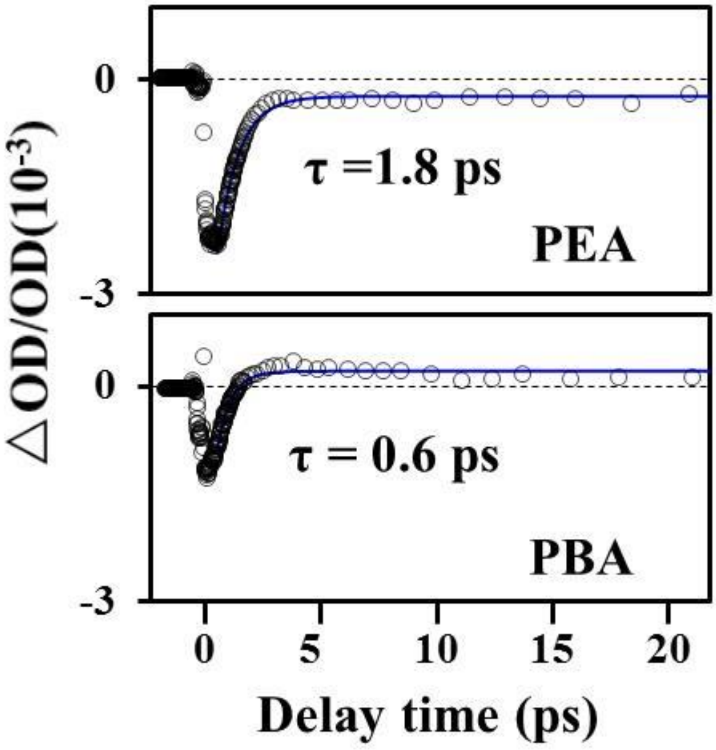

Figure S2. Dynamics of hot electron of NRs at LSPR band based on transient absorption spectroscopy. The blue curves are fitting plots representing decay time constant of τ.

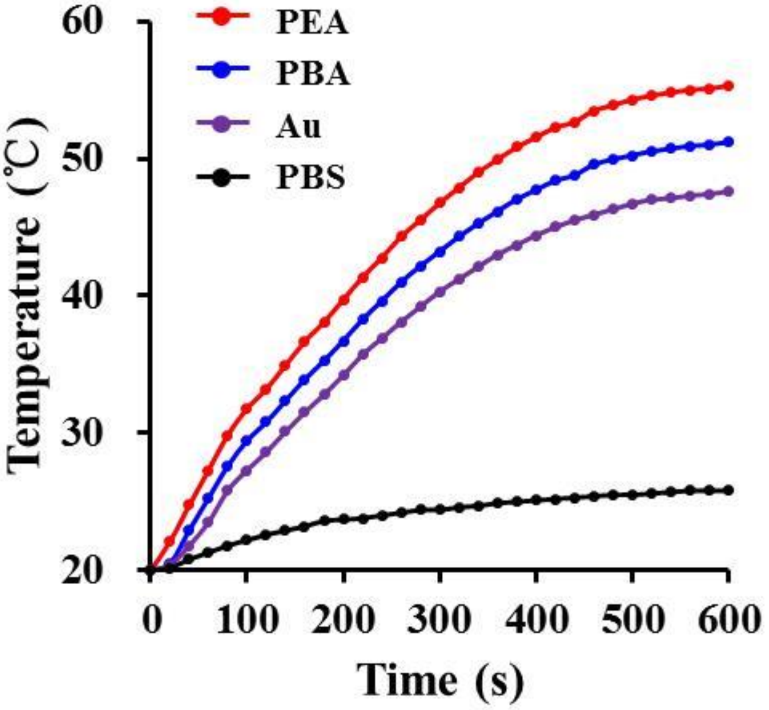

Figure S3. Temperature elevation profiles of PEA, PBA, and Au NRs suspended in aqueous solution (equivalent to 50 μg mL^{-1} Au) irradiated by 808 nm laser (0.75 W cm^{-2}) for 600 s.

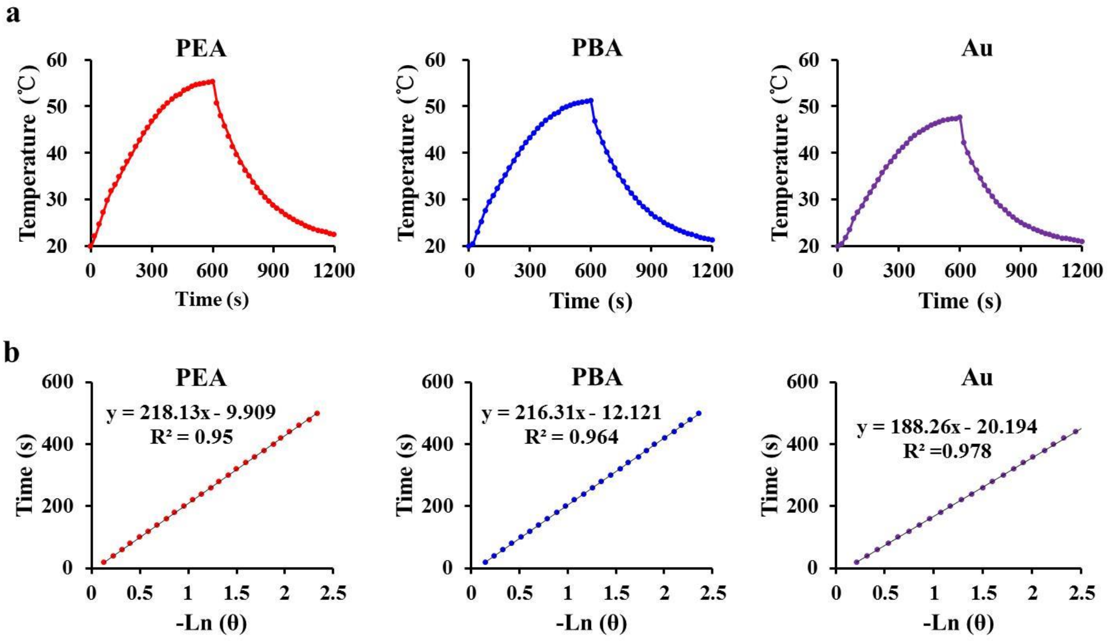

Figure S4. The temperature variation curves (a) and linear regression curves (b) of PEA, PBA, and Au NRs as irradiated by 808 nm laser (0.75 W cm^{-2}) for 600 s, followed by natural cooling with laser light turned off, and determination of the time constant for heat transfer from the system using linear regression of the cooling profiles.

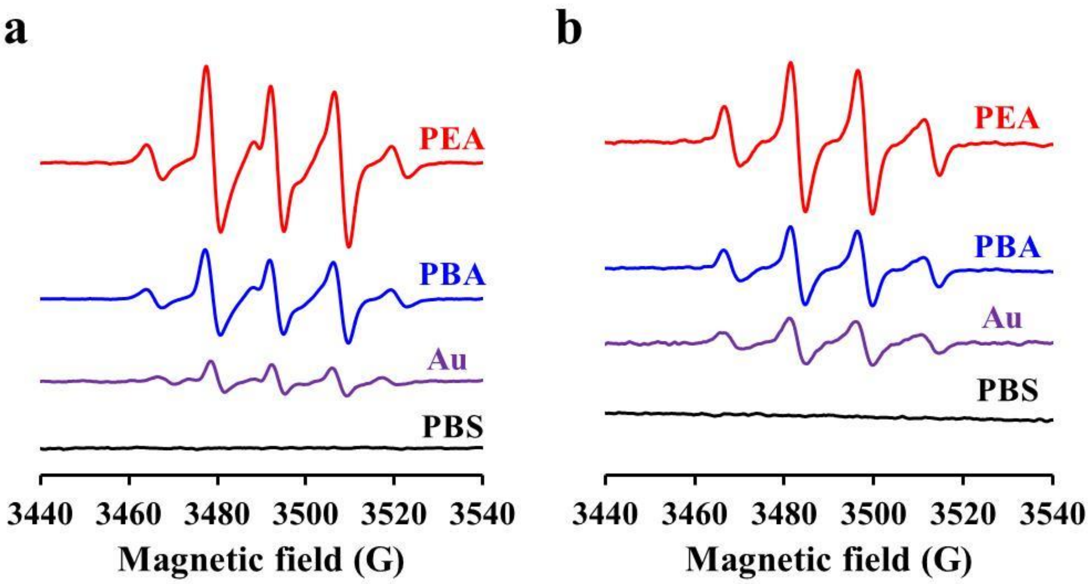

Figure S5. Hot electron-induced photodynamic activity based on ESR analysis. ESR spectra of two spin adducts were generated by Au, PBA, and PEA NRs (equivalent to 25 μg mL^{-1} Au) under 808 nm laser irradiation (0.75 W cm^{-2}) for 10 min. (a) $O_2^{\bullet-}$: DMPO-OOH adduct in DMSO containing 0.2 mol L^{-1} DMPO; (b) •OH: DMPO-OH adduct in aqueous solution containing 0.1 mol L^{-1} DMPO.

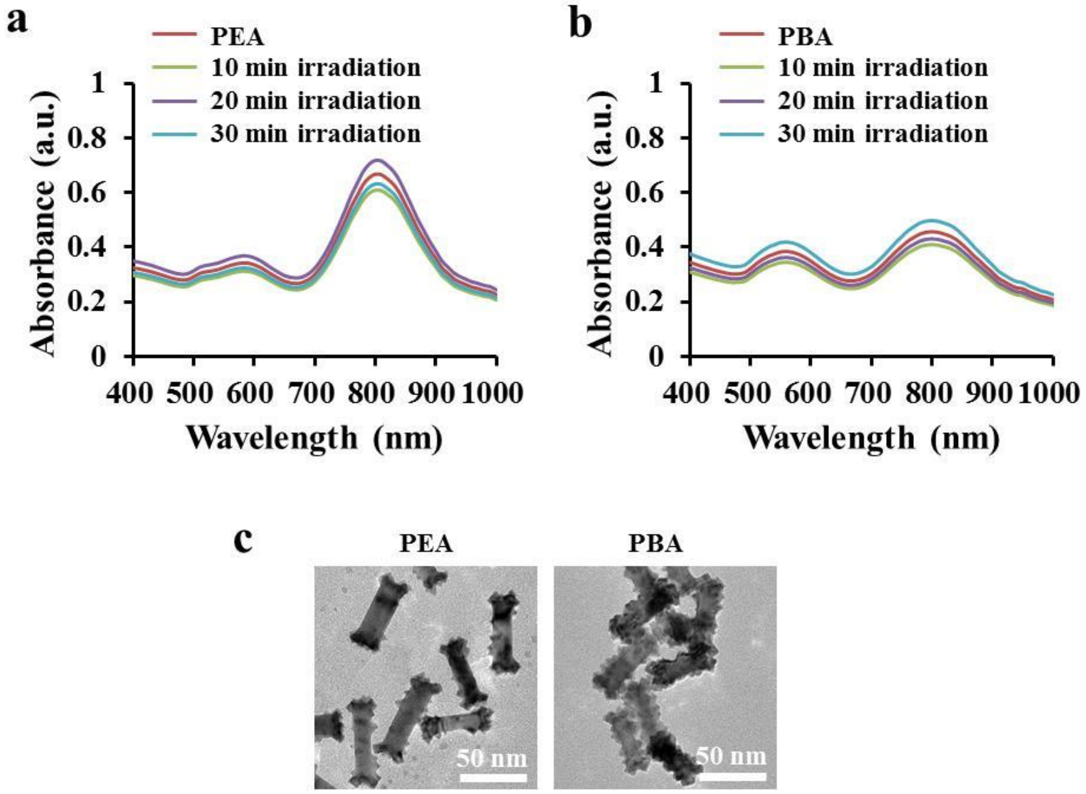

Figure S6. The photostability of PEA and PBA NRs. UV-Vis-NIR absorption spectra of PEA (a) and PBA (b) NR aqueous suspension before and after 808 nm laser irradiation at 0.75 W cm^{-2} for different time periods. (c) TEM images of PEA and PBA NRs after 30 min of 808 nm laser irradiation (0.75 W cm^{-2}).

Photostability of PEA and PBA NRs of was examined by their absorbance spectra (Figure S6a and S6b) and morphology profiles (Figure S6c) after NIR laser irradiation. UV-Vis spectra and TEM images of PEA or PBA NRs showed negligible changes after 30 min of irradiation (0.75 W cm^{-2}), demonstrating the NIR laser-induced temperature elevation and ROS production processes cannot affect the stability of PEA and PBA NRs.

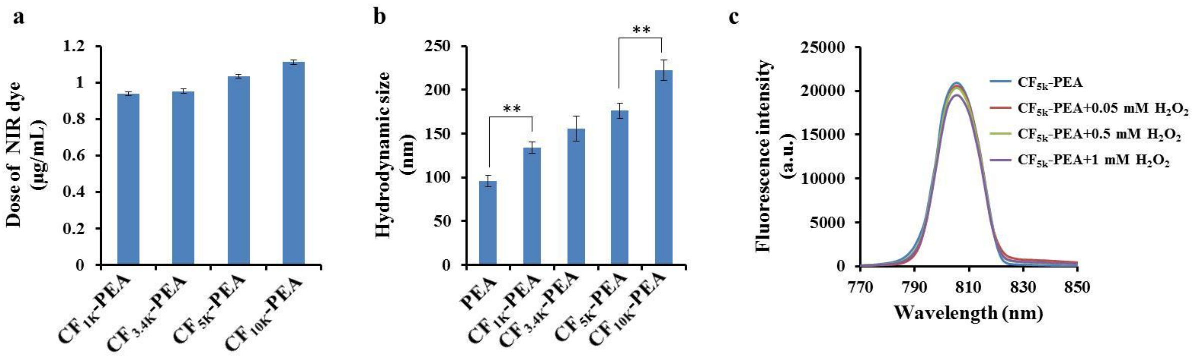

Figure S7. Physicochemical property of $CF_{1\text{-}10k}$-PEA NRs. (a) The content of CF in $CF_{1\text{-}10k}$-PEA NRs. (b) Hydrodynamic size of $CF_{1\text{-}10k}$-PEA NRs in water, **$P<0.01$. (c) NIR fluorescence emission spectra of CF_{5k}-PEA NRs in the presence of various concentrations of H_2O_2.

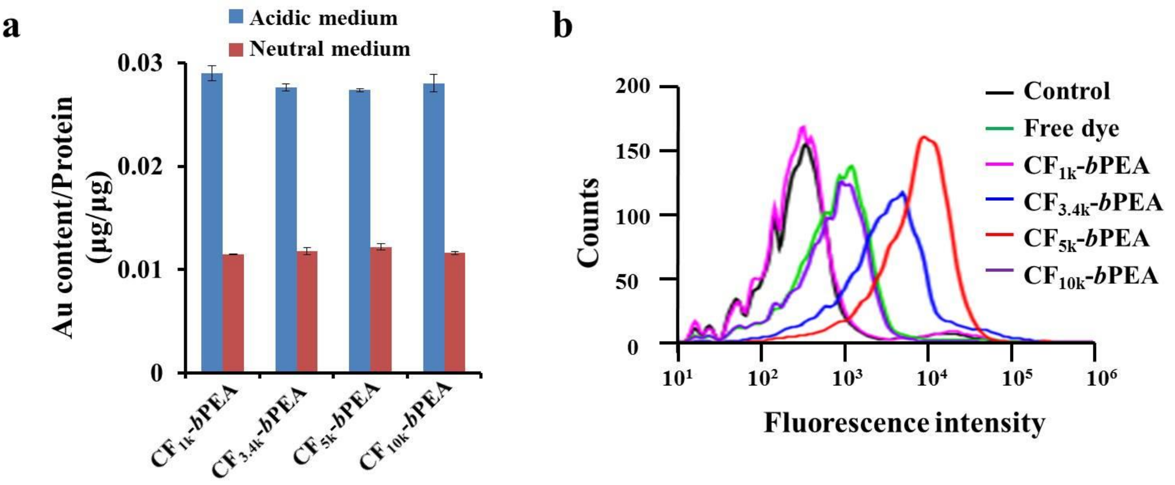

Figure S8. Cellular uptake of $CF_{1\text{-}10k}$-*b*PEA NRs in MCF-7 cells. (a) Cellular Au content analyzed by ICP-OES. (b) Cellular fluorescence intensity analyzed by flow cytometry. Cells were treated with various $CF_{1\text{-}10k}$-*b*PEA NRs (equivalent to 25 μg mL^{-1} Au) for 6 h.

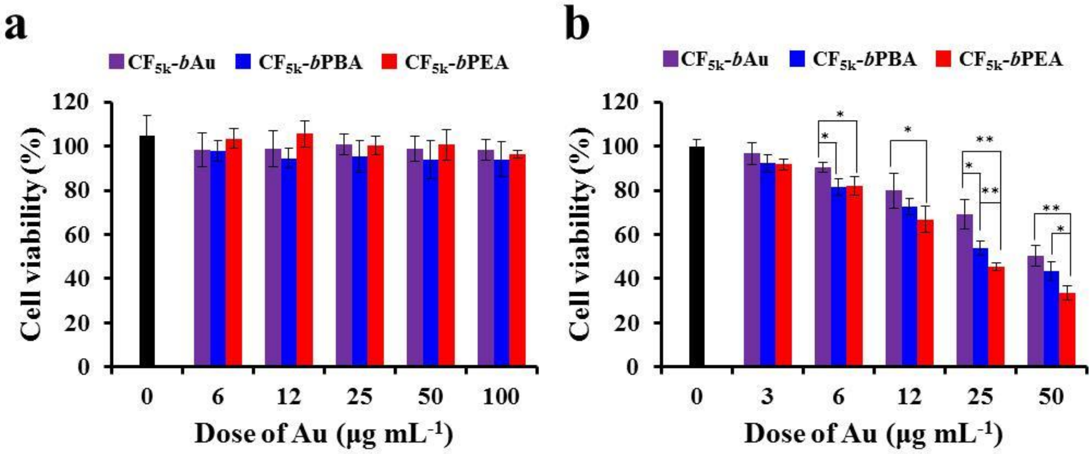

Figure S9. *In vitro* cell viability of CF_{5k}-*b*Au NRs, CF_{5k}-*b*PBA, and CF_{5k}-*b*PEA NRs. (a) MTS viability assessment of MCF-7 cells treated with different concentrations of CF_{5k}-*b*Au NRs, CF_{5k}-*b*PBA, and CF_{5k}-*b*PEA (according to Au content) for 24 h without NIR laser irradiation. (b) MTS viability assessment of MCF-7 cells treated with different concentrations of CF_{5k}-*b*Au, CF_{5k}-*b*PBA, and CF_{5k}-*b*PEA NRs (according to Au content) for 24 h under 5 min of 808 nm laser irradiation (0.5 W cm^{-2}), in normal culture medium, **P*<0.05 and ***P*<0.01.

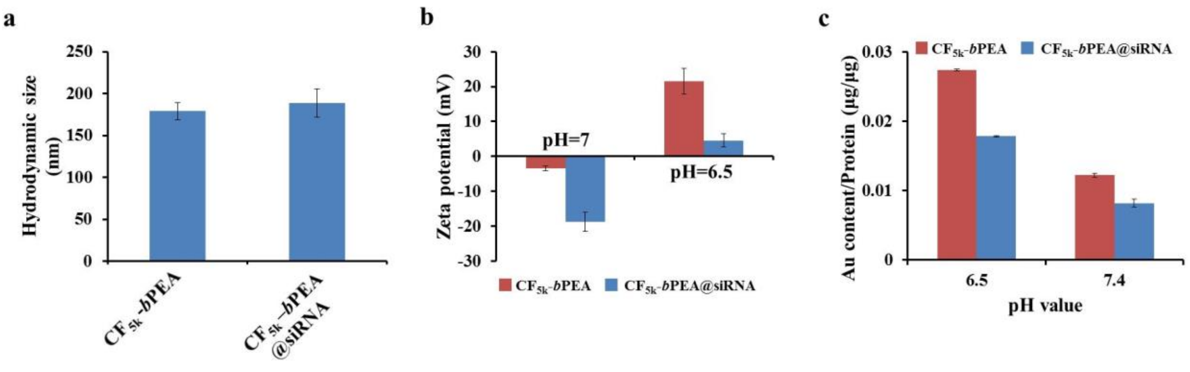

Figure S10. (a) Hydrodynamic sizes and (b) Zeta potentials of CF_{5k}-*b*PEA and CF_{5k}-*b*PEA@siRNA NR aqueous solution. (c) Cellular uptake of CF_{5k}-*b*PEA and CF_{5k}-*b*PEA@siRNA NRs in MCF-7 cells and cellular Au content analyzed by ICP-OES.[5b]

The hydrodynamic size of CF_{5k}-*b*PEA@siRNA NRs did not display a noticeable change compared with that of CF_{5k}-*b*PEA NRs (Figure S10a). The surface charges of both CF_{5k}-bPEA NRs and CF_{5k}-bPEA@siRNA NRs got positive at weak acidic condition (pH=6.5) compared with those under neutral condition (pH=7.4), where CF_{5k}-bPEA@siRNA NRs showed less positive than CF_{5k}-bPEA NRs under weak acidic condition (Figure S10b). CF_{5k}-bPEA@siRNA NRs could also be effectively internalized into MCF-7 cells (Figure S10c).

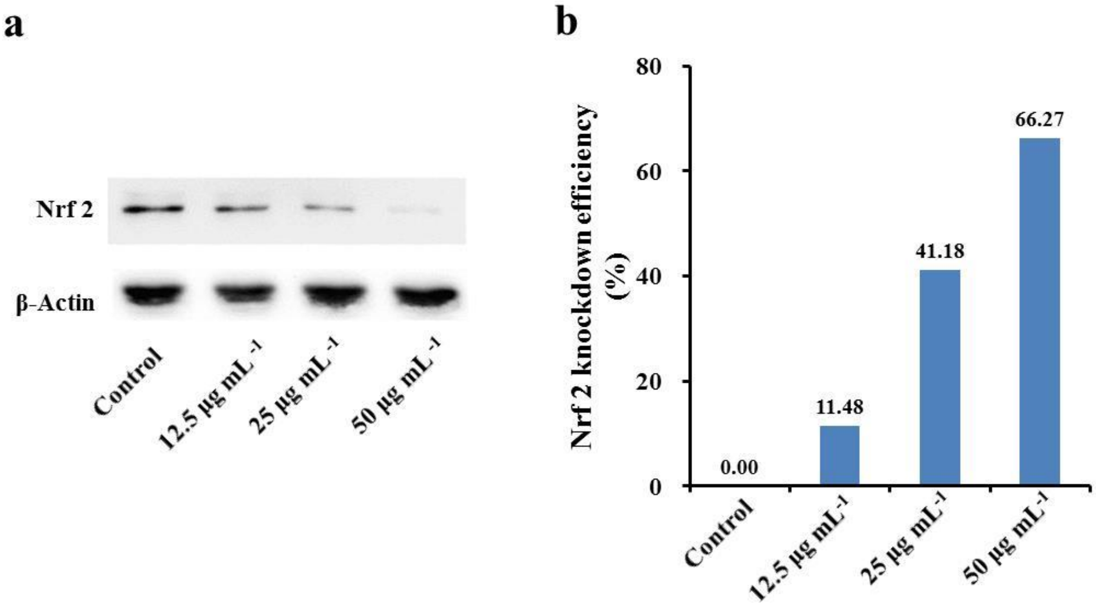

Figure S11. Nrf2 gene knockdown (a) and knockdown efficiency (b) in MCF-7 cells treated with different concentrations of CF_{5k}-*b*PEA@siRNA NRs (according to Au content).

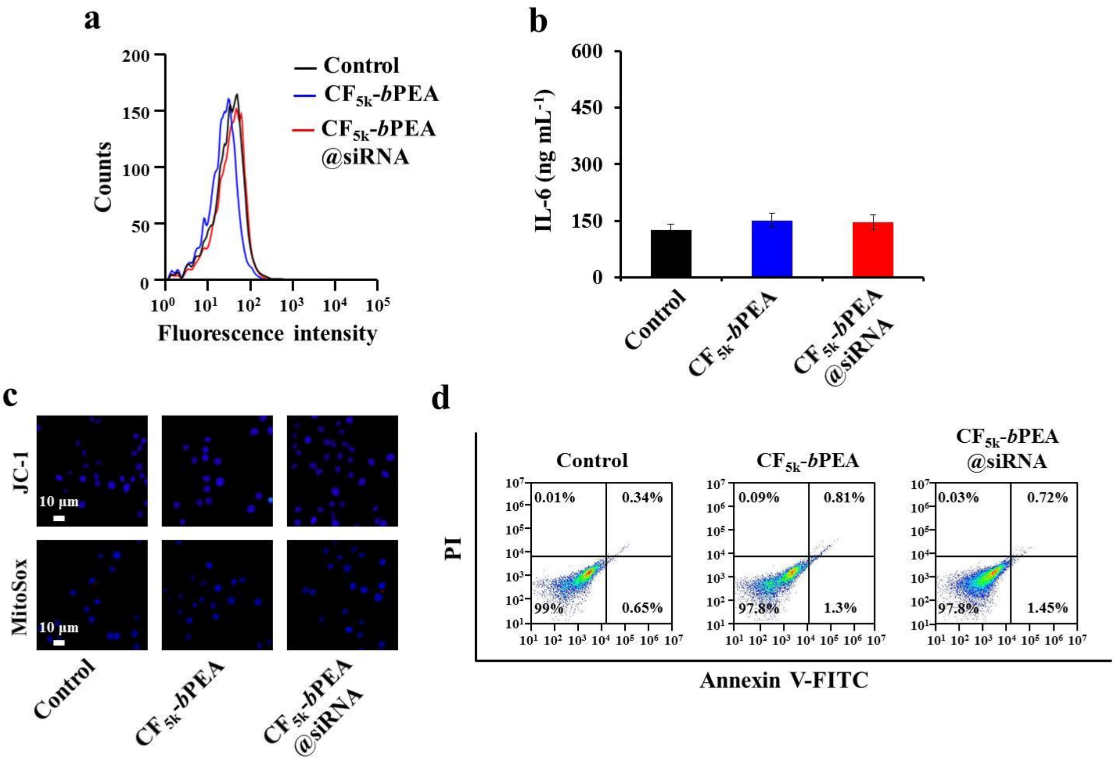

Figure S12. Cellular responses induced by CF_{5k}-*b*PEA and CF_{5k}-*b*PEA@siRNA NRs without NIR laser irradiation. MCF-7 cells were treated with CF_{5k}-*b*PEA or CF_{5k}-*b*PEA@siRNA NRs (equivalent to 25 μg mL^{-1} Au) for 24 h. (a) Flow cytometry analysis of intracellular ROS level based on DCF assay. (b) Elisa assay to assess cellular IL-6 secretion. (c) Fluorescence microscopy images of cells stained by JC-1 or Mitosox Red to detect membrane depolarization or mitochondrial superoxide generation. (d) Flow cytometry analysis of apoptotic cells based on Annexin V-FITC/PI assay.

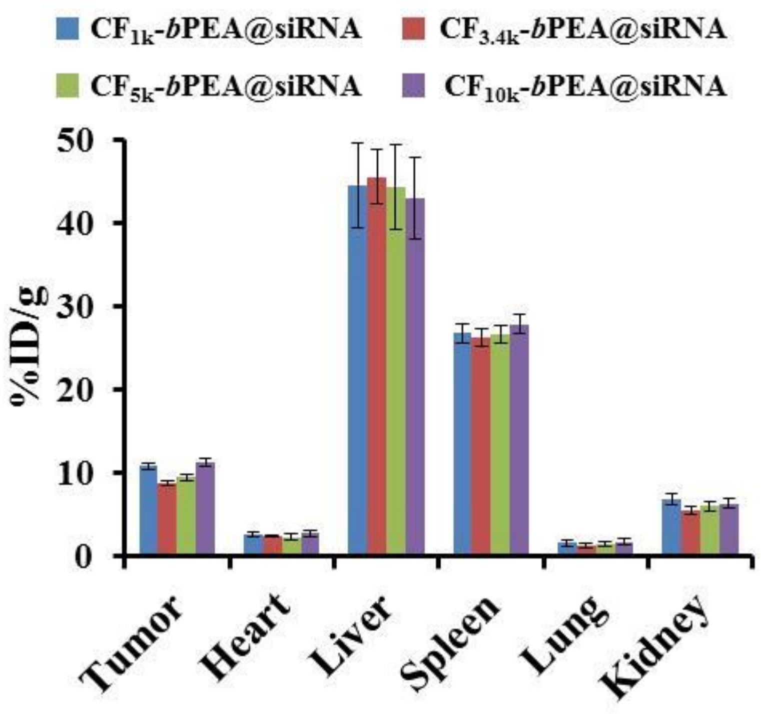

Figure S13. Biodistribution of CF_{1-10k}-*b*PEA@siRNA NRs in MCF-7 tumor-bearing mice at 24 h post-administration based on Au element analyzed by ICP-OES. Data expressed as percentage of the injected dose per gram of tissue (%ID/g).

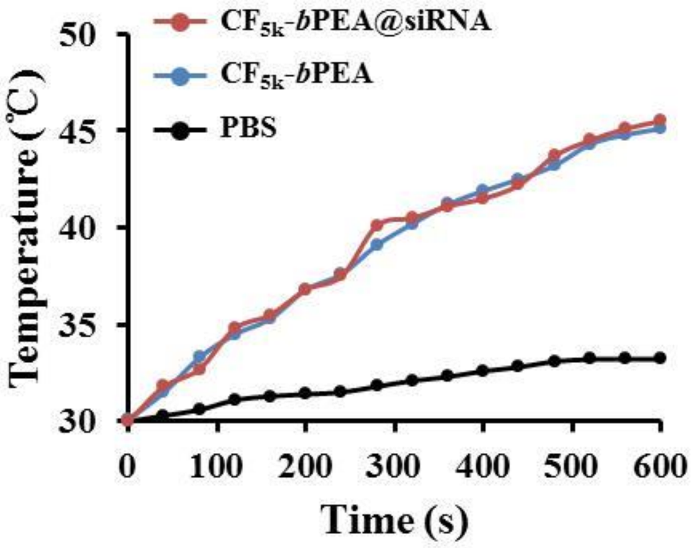

Figure S14. Temperature variation of tumors of MCF-7 tumor-bearing mice treated with CF_{5k}-*b*PEA or CF_{5k}-*b*PEA@siRNA NRs. Mice were intravenously administered with CF_{5k}-*b*PEA or CF_{5k}-*b*PEA@siRNA NRs (equivalent to 20 mg Au/kg mouse). After 24 h, tumor region was irradiated with an 808 nm laser for different time periods at a power density of 0.75 W cm^{-2}.

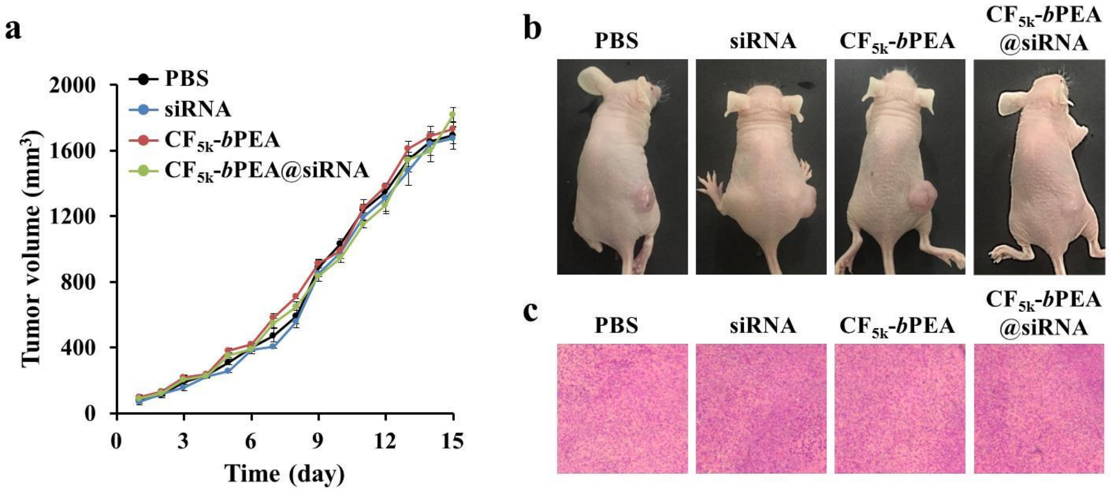

Figure S15. *In vivo* therapeutic effects of CF_{5k}-*b*PEA and CF_{5k}-*b*PEA@siRNA NRs without NIR laser irradiation. (a) Tumor growth curves of MCF-7 tumor-bearing mice intravenously administered with PBS, siRNA, CF_{5k}-*b*PEA NRs, and CF_{5k}-*b*PEA@siRNA NRs (equivalent to 20 mg Au/kg mice or 15 μmol L^{-1} siRNA/kg mice) without NIR laser irradiation. (b) Representative photos of MCF-7 tumor-bearing mice at the end of treatment. (c) H&E stained tumor sections at the end of treatment.

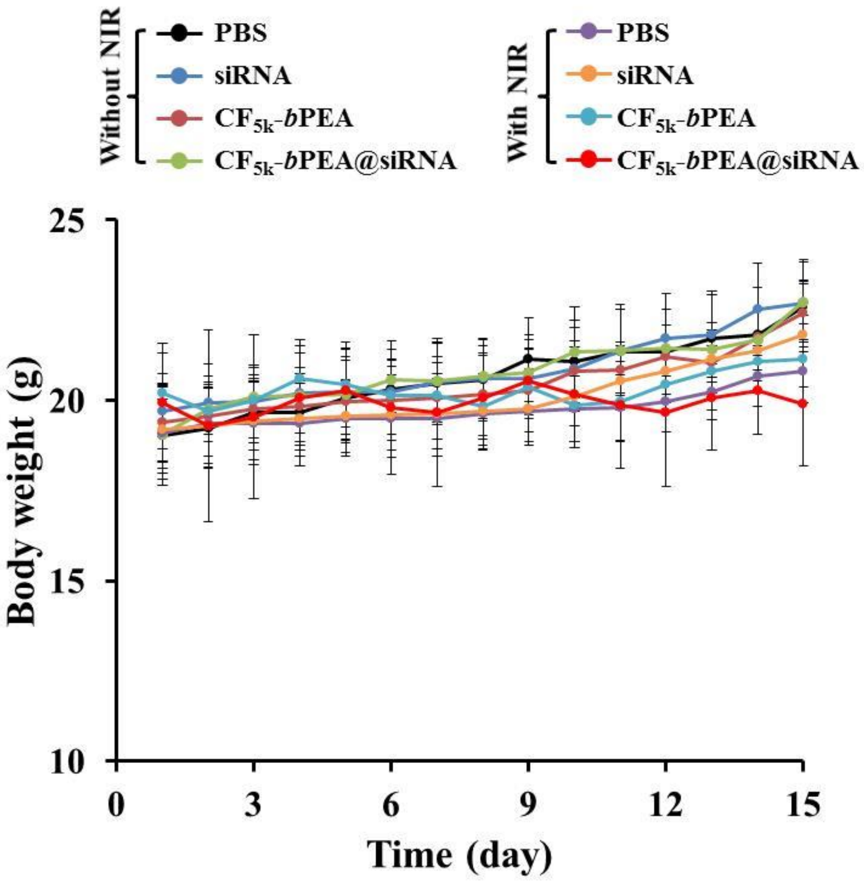

Figure S16. Body weight variation of MCF-7 tumor-bearing mice during the treatment period. Mice were intravenously administered with PBS, siRNA, CF_{5k}-*b*PEA NRs, and CF_{5k}-*b*PEA@siRNA NRs (equivalent to 20 mg Au/kg mice or 15 μmol L^{-1} siRNA/kg mice). At 24 h post-administration, the tumor region of mice was irradiated by an 808 nm laser for 10 min at a power density of 0.75 W cm^{-2} or not. Body weights of mice were recorded every day within 15 days.

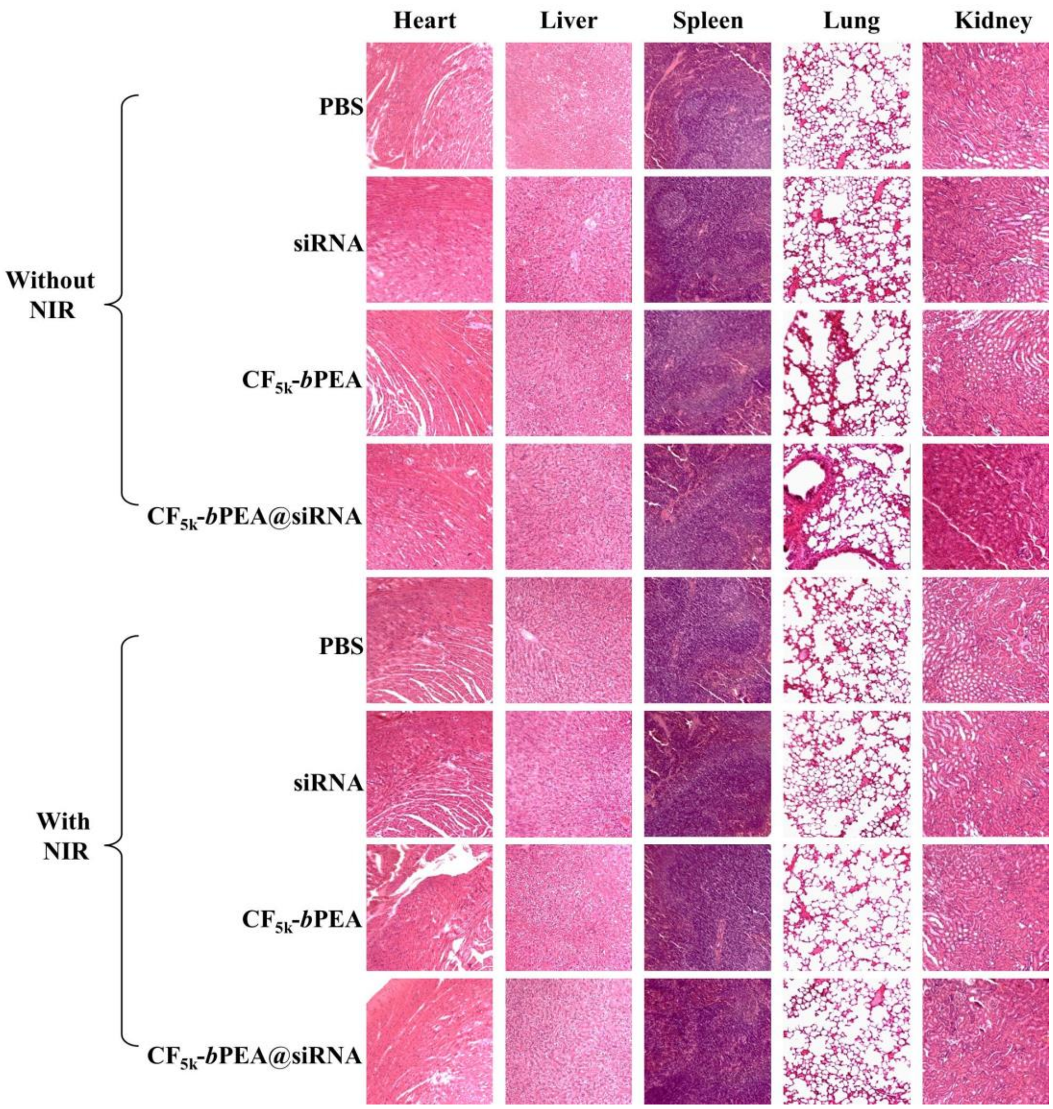

Figure S17. H&E stained histological images of major organs collected at the end of treatment.

Reference:

[1] M. Li, S. K. Cushing, N. Wu, *Analyst* **2015**, *140*, 386.

[2] G. Sun, J. B. Khurgin, C. C. Yang, *Appl. Phys. Lett.* **2009**, *95*.

[3] K. A. Kang, J. Wang, J. B. Jasinski, S. Achilefu, *J. Nanobiotechnology* **2011**, *9*, 16.

[4] K. Nozaki, S. Kita, T. Baba, *Opt. Express* **2007**, *15*, 7506.

[5] Y. C. Tsai, C. F. Lin, J. W. Chang, *Optical Review* **2009**, *16*, 347.
